# Inductively Coupled Plasma-Mass Spectrometry (ICP-MS), a Useful Tool in Authenticity of Agricultural Products’ and Foods’ Origin

**DOI:** 10.3390/foods11223705

**Published:** 2022-11-18

**Authors:** Eleni C. Mazarakioti, Anastasios Zotos, Anna-Akrivi Thomatou, Achilleas Kontogeorgos, Angelos Patakas, Athanasios Ladavos

**Affiliations:** 1 Department of Food Science and Technology, University of Patras, 30100 Agrinio, Greece; 2 Department of Sustainable Agriculture, University of Patras, 30100 Agrinio, Greece; 3 Department of Agriculture, International Hellenic University, 57001 Thessaloniki, Greece

**Keywords:** ICP-MS, geographical origin, authenticity, traceability, agricultural products, foods, beverages

## Abstract

Fraudulent practices are the first and foremost concern of food industry, with significant consequences in economy and human’s health. The increasing demand for food has led to food fraud by replacing, mixing, blending, and mislabeling products attempting to increase the profits of producers and companies. Consequently, there was the rise of a multidisciplinary field which encompasses a large number of analytical techniques aiming to trace and authenticate the origins of agricultural products, food and beverages. Among the analytical strategies have been developed for the authentication of geographical origin of foodstuff, Inductively Coupled Plasma Mass Spectrometry (ICP-MS) increasingly dominates the field as a robust, accurate, and highly sensitive technique for determining the inorganic elements in food substances. Inorganic elements are well known for evaluating the nutritional composition of food products while it has been shown that they are considered as possible tracers for authenticating the geographical origin. This is based on the fact that the inorganic component of identical food type originating from different territories varies due to the diversity of matrix composition. The present systematic literature review focusing on gathering the research has been done up-to-date on authenticating the geographical origin of agricultural products and foods by utilizing the ICP-MS technique. The first part of the article is a tutorial about food safety/control and the fundaments of ICP-MS technique, while in the second part the total research review is discussed.

## 1. Introduction

**Figure 1:**
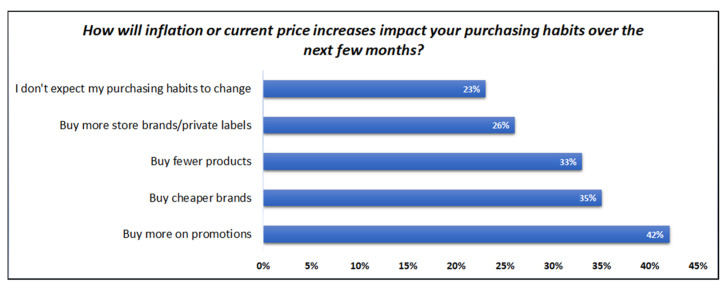
Impact of purchasing decision of consumers due to inflation or higher prices. A higher percentage (80%) of the consumers would choose at least one of the above. Source: Ipsos Coronavirus Consumer Tracker, fielded 4–5 January 2022, among 1600 U.S. adults [2].

Food safety management (or food forensics) focuses on the strengthening of food safety and quality control procedures [1]. Effective control protocols shield health and well-being of people, and subsequently support financially domestic, regional, and international markets and producers. The design of food safety and quality control systems has become more essential than ever due to the tendency to low-cost products. Global financial crisis, COVID-19 pandemic and plenty of other social issues have resulted in economic fallout. Due to lockdowns millions of people suffer a lot of hardship among others unemployment, shortages in supply chain, higher transportations expenses which directly rise product’s final price. The increased cost of living due to inflation has influenced the purchasing decision of consumers, most of the times against the quality. The chart flow in Figure 1 presents the change in purchasing behavior of consumers in United States [2].

According to World Health Organization (WHO), more than 200 diseases are spread around the world, through food contaminated with infectious microorganisms, viruses or chemical substances [3]. Food contamination could be caused in any stage of the supply chain process; however, knowing the geographical origin of edible products consists the front line of quality control. Due to this fact, consumers have become more cautious about the origin of foodstuff hence, they require for supply chain transparency. In addition, given the tremendous global demand for food, some producers debase the quality of their products by either altering the components or by mislabeling the products for economic gain. Frequently, authentic products are partially or totally substituted by undeclared ones, with the intention of reducing the cost production [4]. Noticeable fraudulent activity has been reported in agri-food industry focusing in many cases on rice [5,6], wine [7], milk [8] and olive oil [9,10].

All developed countries have enacted food laws laying down the basic principles and procedures for safety and quality. American Viticultural Areas in United States, Wine of Origin in South Africa, Denominación de Origen in Spain and Denominazione di Origine Controllata in Italy are few of the standardization organizations around the world supervising the production process and certifying the origin of agricultural products, foods and beverages. In an attempt to minimize and/or prevent food adulteration, in 2002, European Union (EU) adopted the Regulation (EC) No 178/2002 (amended by Regulations (EU) No. 652/2014, No. 2017/228 and 2019/1381) and established the European Food Safety Authority (EFSA). The latter is an independent scientific agency which is responsible for examining novel issues and hazards, and simultaneously developing the evaluation methodologies providing scientific advice upon request of EC and EU Member States. Particularly, EU has established regulations which define the obligation of indicating the origin of primary ingredients (Regulation (EU) No 1169/2011 and Implementing Regulation (EU) 2018/775), the origin of meat (Regulation (EU) No 1337/2013), and the origin of fishery and aquaculture products (Regulation (EU) No 1379/2013). Additionally, the EU traceability regulations (Regulation (EC) No 178/2002, Regulation (EU) 1151/2012 amended by Regulation (EU) 2017/625) protects the names and the reputation of agri-food products from specific geographical provenance as follows:

(a) Protected designation of origin (PDO/food, agricultural products, and wines). All the production stages are taken place in specific geographical region.
(b) Protected geographical indication (PGI/food, agricultural products, and wines). At least one of the production stages are taken place in specific geographical region.
(c) Geographical indication (GI/Spirit drinks, and aromatized wines). In this category, at least one stage of the production should be in a specific area.

It is more than obvious that authenticity and traceability provide the base on quality control and safety of agricultural products. The last decades, much effort has been focused on developing highly sophisticated biological and chemical scientific methods for certifying the origin and authentic nature of agri-food [11]. Generally, biological methods aim to evaluate the organic part (i.e., DNA, sugars, lipids, and proteins) while determination of multi-elemental compositions and/or isotopic fingerprint of an agricultural product reflects the growth conditions on a particular geographical area. Among the traceability methods, Nuclear Magnetic Resonance (NMR) is typically used for analyzing the chemical and molecular food composition. NMR spectroscopy or in combination with other techniques have been applied to determine the origin of foodstuffs [11]. Trace and rare earth elements have also been shown to play an important role on geographical origin and authenticity of a variety of products including, among others, wheat grains, wines, dairies, olive oils, legumes and meats. Currently, atomic and mass spectrometry which comprise Atomic Absorption Spectrometry (AAS), Atomic Fluorescence Spectrometry (AFS), Flame Atomic Absorption Spectrometry (FAAS), Inductively Plasma Optical Emission Spectrometry (ICP-OES), Inductively Coupled Plasma Atomic Emission Spectroscopy (ICP-AES), and Inductively Coupled Plasma Mass Spectrometry (ICP-MS) are the most common analytical methods for the elemental or multi-elemental determination of food samples [12].

Isotope-Ratio Mass Spectrometry (IRMS) is able to provide a database of stable isotope ratios of C (^13^C/^12^C), N (^15^N/^14^N), O (^18^O/^16^O), H (^2^H/^1^H) and S (^34^S/^32^S). These elements are directly affected by the growth environment of the product, as they participate in all significant biological and ecological pathways. Hence, they can provide a stable isotope fingerprinting representative of a specific geographical provenance which has obvious advantages [13]. Nonetheless, it has been noticed that concentrations of light elements (C, O, N, H and S) are occasionally influenced by their involvement in biological and ecological cycles which is a negative aspect of traceability [14,15]; therefore, isotope ratios of heavy elements (Sr and Pb) are also investigated. Sr and Pb could be considered as good candidates for trace markers as the isotope ratios of each element (^87^Sr/^86^Sr, and ^206^Pb/^204^Pb, ^206^Pb/^207^Pb and ^208^Pb/^206^Pb) are associated with the age of the substratum [16,17,18]. Assuming that the amount of these elements on the soil are proportionally related to their concentrations absorbed by the plant, they could be excellent markers to predict the geographical origin. In case of Lead, the anthropogenic source (fertilizers, pesticides etc.), which leads to environmental pollution, should also be considered.

As an alternative or complementary to C, N, O, H and S stable isotope analysis, multi-elemental composition analysis is increasingly being investigated in identifying the geographical origin of edible products. In the same manner as with the latter elements, the composition of metals in agri-food substances, is directly related to their availability and mobility in the soil of the growing area. Considering the elemental diversity of the different substrata, multi-elemental analysis of agricultural products can lead to provenance recognition. Several research studies utilize ICP-MS and/or IRMS techniques to trace the origin and certify the authenticity of food products. Comparing with other analytical methods ICP-MS is a precise and accurate technique with wide measurement range (for more than 65 elements), low detection limits (ppt), large capacity sample, and developed methods which can minimize the possible interferences. Furthermore, it is able to perform isotopic analyses of heavier elements than the previously mentioned, broadening the employment of isotope ratio method [19]. Ignacio Garcia Alonso and co-workers published in 2022, an excellently written and well explained tutorial review about the use of ICP-MS for isotopic measurements [20]. Overall, ICP-MS finds application in disciplinary areas of research, including pharmaceuticals, medicine, food chemistry, environmental science, and semiconductors.

The present systematic review aims to gather the research work has been done to date, regarding the application of ICP-MS as unique technique or as complementary to stable isotope analysis in authenticity of geographical origin of agri-food products.

## 2. ICP-MS: Understanding the Basic Principles

**Figure 2:**
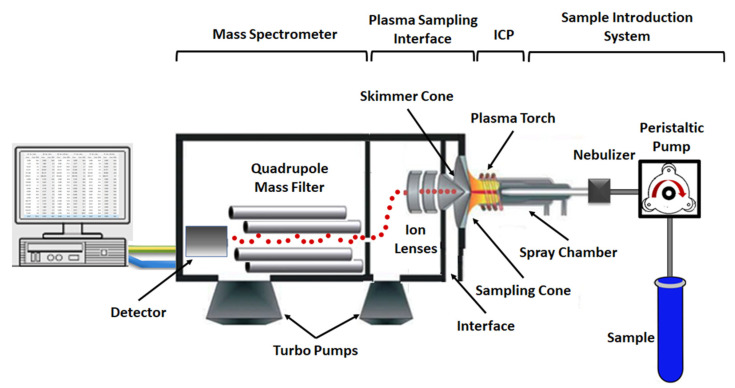
Schematic diagram of the main components of an ICP-MS (Figure adapted with permission and modified from Ref. [21]).

ICP-MS is a robust analytical technique for the determination of multi-elemental composition (qualitatively), concentration (quantitatively) and isotopic abundances of various matrices. Generally, an ICP-MS instrument consists of (i) a sample introduction system, (ii) an ion source (Inductively Coupled Plasma, ICP), (iii) the electrostatic lenses, (iv) an interface, (v) a mass spectrometer and (vi) a detector. Figure 2 depicts a typical schematic representation of the basic components of ICP-MS.

In food chemistry, prior to analysis, sample preparation procedure comprises various steps depending on material’s physical state which could include washing, lyophilization, crushing, extraction, and homogenization. In all cases a thermally-assisted digestion in acidic conditions (HNO_3_, HNO_3_/H_2_O_2_, HNO_3_/HCl, or HNO_3_/HF) is essential. According to the literature, samples are commonly digested with pure HNO_3_ (65–70%) on microwave oven, followed by dilution of the resulting solution with ultra-pure water [22]. It is an essential step of the analysis leading to the dissociation of sample’s matrix and the simultaneous release of the elements (analytes).

Due to the fact that initially the ICP-MS analysis was designed for liquids, samples are ordinarily pumped into a solution nebulizing system in liquid phase. Although, gases and solids are able to be inserted by using diverse introduction systems including chemical gas generation, electrothermal vaporization, high performance liquid chromatography (HPLC) or laser ablation [23]. In a typical arrangement, samples are placed into an autosampler and a peristaltic pump guides them to the nebulizer. Each sample is mixed with argon (Ar) gas creating an aerosol which enters to the spray chamber. The later extracts the large aerosol droplets, due to the inefficiency of the plasma to dissociates them, and subsequently normalizes the flow of the liquid coming from the peristaltic pumps [24,25]. Thereafter, the final fine aerosol traverses the main channel of the argon plasma. The high temperature plasma fosters the ionization of the sample by vaporizing, decomposing, atomizing and finally ionizing the droplets of the aerosol. It should be noted that the ionization potential and the temperature of the plasma define the degree of ionization of the elements [26]. Argon has much higher ionization potential than the majority of the elements, leading to their efficient ionization and creating singly charged positive ions. The generated positively charged ions are separated from the plasma into the interface region through the orifice (~1 mm) at the tip of the sample cone, which, in turn passes through the next orifice (~0.45 mm) of the skimmer cone. Between the two cones the interface pressure is constant at 150–300 Pa. Skimmer cone is the entrance of the high vacuum area (~77 × 10^−5^–1 × 10^−3^ Pa) where the ion lenses and the mass analyzer are located. The electrostatic lenses or ion optics focus and direct the ion beam toward the mass spectrometer, and simultaneously redirect unwanted species (i.e., non-ionized substances and photons) which are the main reason of background noise and signal instability when they are observed by the detector. The fundamental operation of the lenses depends on the manufacturer company of the ICP-MS instrument (i.e., Agilent, and Perkin Elmer). Following the procedure, the ion beam reaches the mass analyzer, the type of which is depended on the ICP-MS system. Generally, in food analysis, quadrupole or octupole type mass analyzer is used. The function of all mass analyzers is based on the determination of ion’s mass/charge (*m/z*) ratio, which is responsible for the division of ions in a sample [27,28]. At the end of mass analyzer, the positively charged ions, separated according to their *m/z* ratio, strike the detector, which is largely an electron multiplier (EM). The resulting culminated signal pulse is referred as ion ‘count’ (with units counts per second (CPS)) and corresponds to ions with specific *m/z* ratio. Due to this fact, ICP-MS is a highly sensitive analytical technique with detection limits being in ng/L for most of the elements.

**Figure 3:**
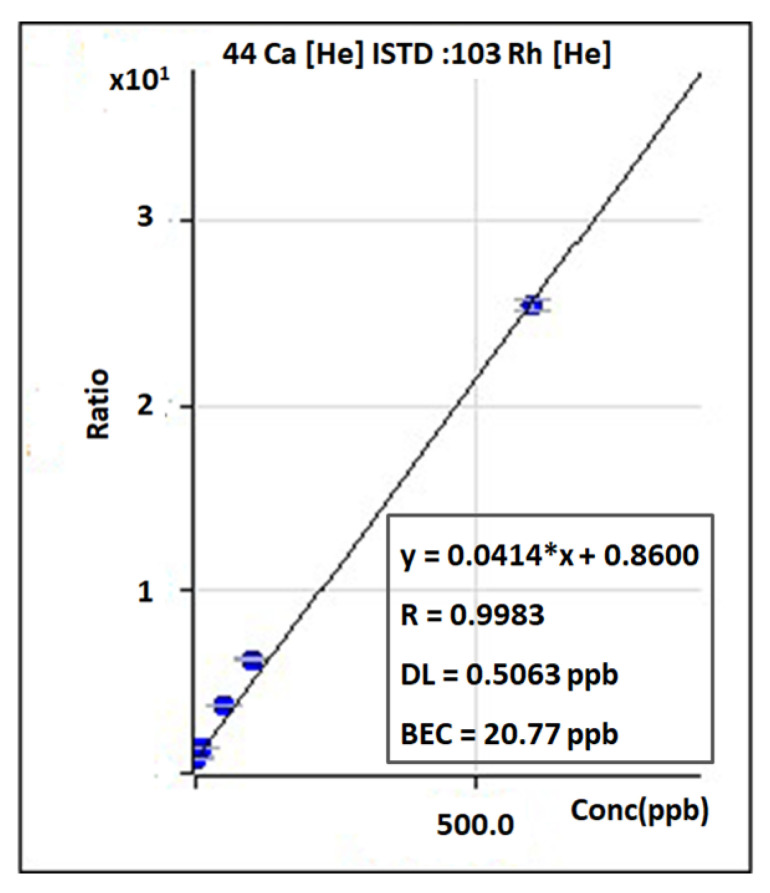
Example of a typical calibration standard curve (^44^Ca in helium mode ([He]) with Rh internal standard). y = 0.0414x + 0.8600: The output equation extracting after fitting the data to a linear regression, R: Standard Deviation, DL: Detection Limits, BEC: Background Equivalent Concentration. Units of Ratio between the signal of ^44^Ca and ^103^Rh (*y*-axis): counts per second (CPS).

**Figure 4:**
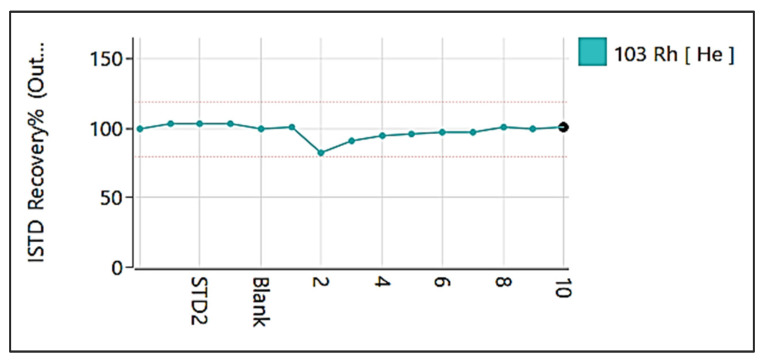
Plot of internal standard stability in an ICP-MS analysis of agri-food samples. Y-axis is referred to the percentage recovery of internal standard (Rh) solution.

As most analytical techniques, ICP-MS utilizes a calibration curve to convert the CPS signal to concentration. Calibration curves are constructed, for each element which is needed to be investigated, by measuring solutions of known concentrations. Figure 3 depicts an example of a calibration curve for calcium (^44^Ca) measured in different concentrations (2, 10, 50, 100, and 600 ppb). Moreover, in an attempt to correct variations in instrument’s sensitivity and/or matrix effects internal standardization (IS) technique is performed. One or more internal standard (IS) elements with similar masses and ionization potentials with the measured analytes are used in order to monitor the analyte-IS signal ratio [28,29,30]. When this ratio is relatively constant the (±20% referring to IS recovery, Figure 4) alterations in operational conditions and matrix effects are minimized, improving the sensitivity and precision of the ICP-MS analysis. Depending on the material which is analyzed other correction methods have been reported including matrix-mached calibration [31,32], method of standard additions [33] and isotope dilution [34,35].

Interferences in ICP-MS analysis are divided into two main categories: (a) spectroscopic, when atomic or molecular ions have equal m/z ratio as the analyte, and (b) non-spectroscopic, which occurred by sample matrix and/or instrument drift. Spectroscopic interferences are potentially a result of isobaric elements (i.e., ^114^Cd^+^ and ^114^Sn^+^), double charged (i.e., ^88^Sr^2+^ and ^44^Ca^+^), or polyatomic ions (i.e., ^44^CO_2_^+^ and ^44^Ca^+^), and/or tailing interferences due to overlapping of two adjacent peaks in the spectra. Table 1 lists a number of the most common spectroscopic interferences [28,36]. On the other hand, analytical errors may be occurred by the non-spectroscopic interferences. The latter arise from matrix effects or instrument drifts leading to suppression or enhancement of the signal. Malfunctions at introduction system (Sample Introduction Effects), ionization in the plasma (Plasma Effects), and electrostatic phenomena among the ions in the interface and ion lenses (Space-Charge Effects) constitute the origin of matrix effects. On top of that, gradually by the use of instrument salts dissolved in the aerosol deposit around cones. The fact that decreases the size of the orifice of the cones suppressing the analyte’s signal. In-depth study about ICP-MS interferences and various strategies to reduce or eliminate them can be found on the review article written by Wilschefski and Baxter, published in 2019 [28].

According to the literature various methods of data analysis are used for building analytical models in order to accurately determine the geographical origins of the samples based on the concentrations of measured analytes. Principal component analysis (PCA), Cluster analysis (CA), Linear Discriminant Analysis (LDA), and Canonical Discriminant Analysis (CDA) consist the most common analytical models [5].

## 3. Database and Literature Search

**Figure 5:**
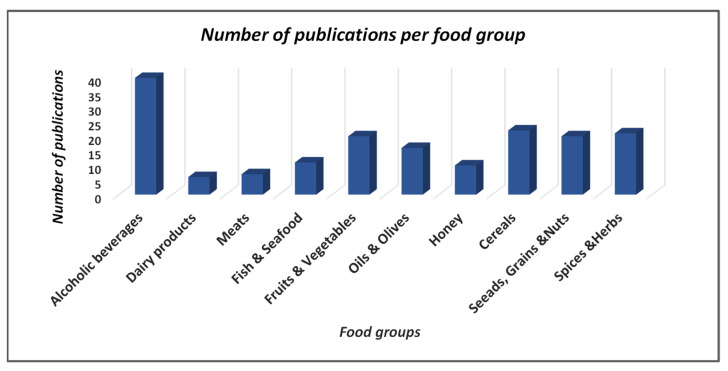
Diagrammatic representation of the number of publications with regards to the type of product.

Two online databases, Web of Science (Clarivate) and Scopus (Elsevier), were used for the literature searching. The terms were used, initially, on each of the above databases, are the following: *ICP-MS* AND authentication origin, ICP-MS AND geographical origin, ICP-MS AND geographical fingerprints, ICP-MS AND protected geographical indication, ICP-MS AND protected designation of origin, ICP-MS AND traceability, ICP-MS AND geographical origin AND agricultural products, ICP-MS AND geographical origin AND foods, ICP-MS AND protected geographical indication AND agricultural products, ICP-MS AND protected geographical indication AND foods, ICP-MS AND protected designation of origin AND agricultural products, ICP-MS AND protected designation of origin AND foods, ICP-MS AND traceability AND agricultural products, and ICP-MS AND traceability AND foods. As a second step, the type of each individual product (**X**), resulting from the initial literature investigation, was added as a term as follows: ICP-MS AND authentication origin AND (**X**), ICP-MS AND geographical origin AND (**X**), ICP-MS AND geographical fingerprints AND (**X**), ICP-MS AND protected geographical indication AND (**X**), ICP-MS AND protected designation of origin AND (**X**), ICP-MS AND traceability AND (**X**). All the included records are screened to be relevant to our topic of study, which includes the employment of ICP-MS analysis for the geographical origin authentication of agricultural products and foods. Moreover, *Zotero* software was used to prepare the references and simultaneously to avoid duplicated publications. The final 173 articles were classified according to the food group investigated for the geographical discrimination with the use of ICP-MS technique (Figure 5).

## 4. Discussion

Interpretation of ICP-MS analysis to authentication and traceability would be easier in groups of food samples. Therefore, the products were divided into classes with particular features. Our aim is to provide and discuss the most important information for each group of products. Table 2 lists the published studies up to date (August 2022) referring to ICP-MS analysis as a method for geographical origin authentication of agricultural products and foods.

### 4.1. Alcoholic Beverages

Wine belongs to the most strictly protected products worldwide. Fast and accurate analytical methodology for authenticity and traceability has become apparent. It has been shown that elemental analysis (trace and rare elements, and stable isotope ratios) is commonly used for the geographical origin of wines [37].

**Figure 6:**
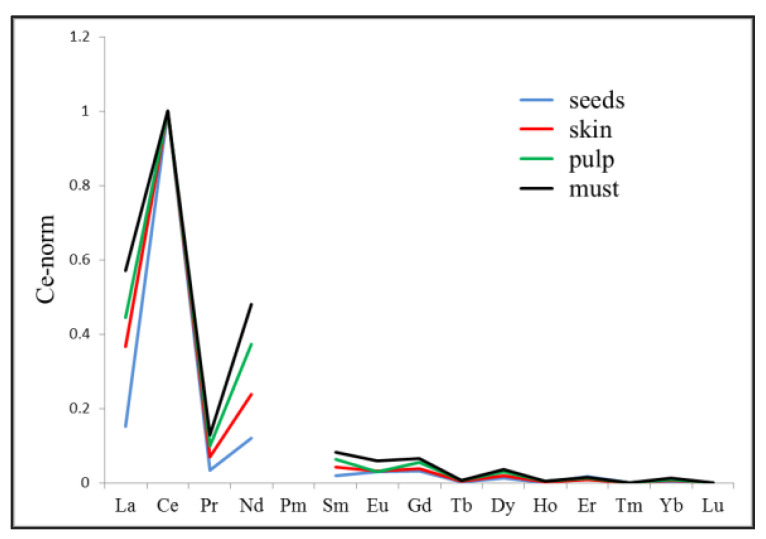
The diagram represents the lanthanide distribution in the various parts of *Primitivo* grapes. Figure is reprinted with permission from Ref. [7]. Promethium (Pm) is not measured as all of its isotopes are radioactive.

In 1997, Greenough et al. [38], performed multi-elemental analysis with the use of ICP-MS, in an attempt to build element ‘fingerprinting’ of different varieties of wine from the Okanagan Valley (British Columbia, Canada). The same year, Baxter and coworkers [39] analyzed the trace element composition of wines from England and Spain. According to our knowledge, the forementioned studies were the first endeavors to distinguish diverse varieties and origins of wines with the employment of ICP-MS. Since then, several studies [40,41,42,43,44,45,46,47,48,49,50,51,52,53,54,55,56,57,58,59] (Table 2) have been carried out on the elemental determination and correlation among various wine varieties, grapes, and soils aiming to trace elements which would be excellent markers for the authentication of geographical origin. In 2021, Wang’s group compared the elemental profile and the variations from soil to grapes and wines from diverse Chinese regions. They claimed that K, Sr, and Li could potentially be used as tracers for geographical origin of Chinese wine; those elements show significant correlation among all the samples [60]. Remarkable results have been shown when multi-elemental analysis is combined with the ^87^Sr/^86^Sr isotope ratio [61,62,63]. ^87^Sr/^86^Sr ratio makes an exceptional marker for the origin determination as it is directly related to the geological age of the bedrock of a territory. Detailed studies by Bora et al. [63], came to the conclusion that there was a relevant geographical origin discrimination of wines based on their elemental composition and ^207^Pb/^206^Pb, ^208^Pb/^206^Pb, ^204^Pb/^206^Pb, and ^87^Sr/^86^Sr isotope ratio. Another notable group of elements which has engaged the scientific attention in food forensics is Lanthanides (Ln) or Rare Earth Elements (REE): Ce, Pr, Nd, Pm, Sm, Eu, Gd, Tb, Dy, Ho, Er, Tm, Yb, and Lu including La, and Y. Samples were analyzed focusing on the REE, claiming that traceability is feasible through soil to grape and must, however changes occurred possibly after clarification process of wine with bentonites [7,64,65,66]. Figure 6 is reproduced by the publication of Aceto et al. [7]; it depicts the variation of lanthanides concentrations measured in pulp, skin, and seed of the grapes and the must. As it is shown only Eu did not follow the stable tendency of the rest lanthanide elements.

Other alcoholic beverages which have been studied by means of multi-element distribution are whiskey [67,68,69], cider [70,71], beer [72,73,74,75] and Chinese liquors [76]. Gajek and co-workers [69] studied extensively diverse varieties and ages of whiskey from different geographical regions and production procedures. Their investigations demonstrated that there were discrepancies in the metals Al, Cr, Cu, Fe, K, Mg, Mn, P, S, Ti, Tl, Zn, and V between single malt and blended Scotch whiskies. Furthermore, it was noteworthy that homemade whiskies from Poland are composed with the highest concentrations of Sr, K, S, and P. They were also observed that Cu, Mn, Zn, and P exhibit alterations in their concentrations during the aging of the samples. In case of beer, earlier this year, Lafontaine et al. [74], published in Food Chemistry their studies on the elemental profile of a wide variety of hops (*Humulus lupulus*) from various territories in German and USA, assuming that the authentication of hops might also be related to the quality and authentication of beer. They concluded that the concentrations of the elements Ba, Ca, Cd, Co, Ni, Mg, Sb, Sr, and U were impacted by the variety and the region of hops. The correlation between the elemental profile of hops and the dry-hopped beer is under investigation. Cider is the less studied alcoholic beverage by means of elemental analysis. In 2007, the group of J. Ignacio García Alonso [70] analyze 67 samples from various regions in order to authenticate their geographical origin. For this purpose, ^87^Sr/^86^Sr isotope ratio and multi-elemental analysis were determined by the combination of ICP-MS and ICP-AES. Fluctuations in the concentration of Na, Mg, Al, K, Ca, Ti, V, Mn, Zn, As, Rb, Sr, Mo, Ba and ^87^Sr/^86^Sr isotope ratio allowed the classification of cider samples originating from Spain, France, England, and Switzerland.

### 4.2. Dairy Products: Milk and Cheese

Trace element analysis in dairy products may derived from the metabolic pathways of the animals and the geographical regions of the farms. In 2008, Benincasa and co-workers [77] investigated the multi-elemental profile of 12 cow and 6 water buffalo milk samples. All the animals were treated equally in the same farm to identify elemental discrimination between the two animal species. Indeed, the authors achieved to differentiate the two types of milk based on their multi-elemental profile. Fernando’s group [8] published, lately, a study which investigated the geographical origin authentication of cow milk from different territories in Sri Lanka by determining the stable isotope ratios of C, H, N, and O as well as the elemental composition with the IRMS and ICP-MS techniques, respectively. It was proposed that a combination of stable isotope ratio of *δ*^15^N, *δ*^13^C (in milk casein), *δ*^15^N, *δ*^18^O, and *δ*^2^H (in whole milk), and the concentration of the metals Li, Al, Cr, Mn, Fe, Co, Ba, and Sr could be promising markers to identify the geographical region of samples which were collected by four different agroclimatic zones. According to the authors the latter elements were possibly related to intrinsic factors of the animal (breed, lactation etc.), dietary habits and supplements. In similar studies, Tedesco et al. (2021) [78], and Aceto et al. (2017) [79] investigated the role of trace and rare earth elements in milk samples, throughout the production chain, from various regions of Italy. It was observed that the concentration of lanthanides remains nearly constant during the milk production and therefore were classified as suitable tracers.

In case of cheese and generally of dairy products, the research is based on the assumption that the metals are not affected by the production procedure; consequently, the resulting records reflect the elemental profile of the geographical region. In 2003, Pillonel et al. [80] analyzed Emmental cheese samples from six European countries for stable isotope ratios (^13^C/^12^C, ^15^N/^14^N, ^18^O/^16^O, ^2^H/^1^H and ^87^Sr/^86^Sr), elemental composition (Ca, Mg, Na, K, Cu, Mn, Mo, I) and radioactive elements (^90^Sr, ^234^U, ^238^U). The concentration of Mo and Na showed interesting variations related to the origin of cheese. In an international collaborative study Camin and co-workers [81] investigated the elemental profile (H, C, N and S stable isotope ratios, and multi-elemental analysis) of seven samples of the hard cheeses Grana Padano and Parmigiano Reggiano according to the IUPAC protocol and ISO Standards 5725/2004 and 13528/2005. Thirteen different laboratories were involved in performing isotope and/or elemental analysis. The determination of Li, Na, Mn, Fe, Cu, Se, Rb, Sr, Mo, Ba, Re, Bi, and U and the H, C, N and S stable isotope ratios were able to verify the origin of both cheeses.

### 4.3. Meats

Soil, feed supplements and environmental pollution are the main sources of elements in meat. In 2005, in a literature review study, Franke and co-workers [82] discussed the methods for the authentication of geographical origin of raw meat by determining the stable isotope ratios and trace elements, concluding that Se and Rb could possibly be used as tracers. In an attempt to authenticate the geographical origin of beef, in 2008, Heaton et al. [83] collected samples produced in various countries worldwide, and analyzed them by using IRMS and ICP-MS techniques. According to the results, they stated that it would be possible to verify the origin of beef particularly based on six key variables *δ*^13^C‰ (defatted dry mass), Sr, Fe, *δ*^2^H‰ (lipid), Rb and Se. Applying Canonical Discriminant Analysis (CDA) a broad classification of samples into European, South American, and Australasian was achieved. Since then, analytical studies, following by the appropriate statistical analysis, have been carried out for examining elements as markers of the geographical origin of yak [84], rabbit [85,86], pork [87], mutton [88], and cured ham [89]. It is worth mentioning that in the latter, the ratio ^87^Sr/^86^Sr has been measured in addition to the elemental composition of the cured ham, since it could be considered as a tracer of the regional geological components. An interesting work has been performed by Meisel’s group in which aimed at developing a method for labeling via REE, unlabeled eggs and poultry products [90], and lamb meat and goat milk [91] by selectively enrich animals feeding with Tb and Tm; those elements have a single stable isotope, and are relatively low-cost. Naturally the composition of lanthanides in food is remarkably low allowing the REE spiking, and simultaneously to secure human’s health. In both cases, the REE labeling method was succeeded allowing the origin determination of the products.

### 4.4. Fish and Seafood

Fish and seafood belong to the most mislabeled foodstuffs around the world. Due to the challenging traceability throughout their production chain, new more effective control methods of origin authentication are developed. ICP-MS tend to become a leading, among others, analytical method for the classification of fish and seafood. The scientific literature revealed that several studies for geographical discrimination and elemental fingerprinting have been performed for sea cucumber [92,93], mussels [94,95], salmon [96], cuttlefish [97], clams [98], crabs [99], sea bass [100], shrimps [101], caviar [102], anchovy products [103] and various marine species from China regions [104].

In 2010, Costas-Rondríguez et al. [94] classified Galician (Spain) and non-Galician mussels from different cultivated areas, by determining their elemental composition combined with different multivariate qualitative methods. Mussels were the first seafood products recognized by European PDO. Sea cucumber has been studied by Liu et al. [92] (2012) and Kang et al. [93] (2018) in an attempt to classify samples from various regions of China. Linear discriminant analysis gave better classification and cross-validation rates on both studies suggested as good tracers the elements Zn, Al, As, Co, Fe, Se and Li, Na, Al, K, Co, Cu, Cd, Sc, respectively. Stable isotope ratio of ^13^C/^12^C, and ^15^N/^14^N and REE (La, Eu, Ho, Er, Lu, and Tb) were investigated as possible markers for the discrimination of different production methods as well as the authentication of geographical origin (Western, Central, and Eastern Mediterranean Sea) of sea bass by Varrà et al. [100], in 2019. Indeed, the combination of the abovementioned analytical methods led to the classification of sea bass samples in relation to production methods and sea regions. In particular, La and Ho resulted to be the elements which were differentiated most in geographical origin classification.

Recently, Varrà et al. [105] reviewed the fish and seafood authentication by inorganic elemental composition.

### 4.5. Vegetables, Mushrooms and Fruits

Vegetables and fruits are products which are directly linked with the soil; thus, it could be an unambiguous correlation between their elemental fingerprints and the geological setting of a region. Traceability and authenticity of vegetable and fruit foodstuffs can be a more straightforward procedure by comparing and relating the elemental distributions of samples and the soils. These days, the authentication of geographical origin of fruits and vegetables is a crucial requirement for food safety, due to the heavy demand for those products which has led to fraudulent labeling practices. Stable isotope analysis (C, N, H, O, S, and Sr) is considered as a pioneer in the field of food forensics. Multi-elemental analysis has been also used, in combination with stable isotope ratio or by itself. Trace elements and lanthanides represent the mineralization characteristics of a particular region.

Reviewing the literature, vegetables such as onion [106,107], garlic [108,109], soybean [110], eggplant [111], various types of pepper [111,112,113], tomato [114,115,116], Chinese cabbage [117], and truffles [118] have been investigated by means of elemental composition for their geographical origin authentication with the use of ICP-MS. Since 2005, Bettinelli et al. [115] investigated the concentration of lanthanide elements of tomato plants and soils from their growing area. They had stated that ICP-MS seems a promising technique for traceability. Almost five years later, Lo Feudo et al. [114] studied tomatoes from Italian farms and tomato paste originating from Italy, California, Greece, and China, resulting in geographical discrimination based on 32 elements as variables. In a similar study, in 2011, the group of Tagarelli determined the elemental fingerprint (25 elements) of the Tropea red onion (PGI brand since 2002), in order to categorize the samples into Tropea and non-Tropea [106]. A noteworthy observation was the contribution of REE and especially of Dy in the authentication of geographical origin of onions. Recently, Segelke et al. [118] published their endeavors to develop an accurate methodology for the geographical discrimination of truffles. Truffle is one of the most expensive products; however, it belongs among to the most misdescribed foodstuffs due to its different varieties which are not easily distinguishable.

**Figure 7:**
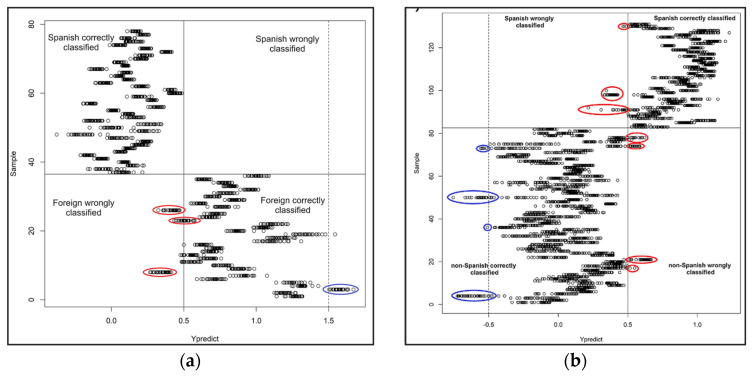
Plots of the PLS-DA for the geographical discrimination of (**a**) mangoes; (**b**) avocados. Figures are reprinted with permission from Refs. [121,122], respectively.

In case of fruits, a series of studies have examined the elemental composition for the authentication of geographical origin of lemons [119], apples [120], mango [121], avocado [122], pears [123], jackfruit [124], and clementines [125]. In 2012, PGI brand Clementine of Calabria were geographically distinguished from non-PGI samples from Spain, Tunisia, and Algeria by Benabdelkamel et al. [125] who developed a multi-element analytical method with the employment of ICP-MS. Similar studies were performed on Italian PGI and non-PGI Turkish lemons by Giorgia Potortì et al. [119]. Muñoz-Redondo et al. published two research projects on the stable isotope and multi-element analysis of avocado [122] and mango [121] for geographical origin authentication purposes. The findings on both reports confirmed that the combination of two techniques lead to more accurate geographical discrimination (Figure 7).

### 4.6. Oils and Olives

The elemental determination of edible oils is a challenging procedure due to their lipophilic matrix, rich in carbon. The low concentration levels of trace and rare earth elements can be detected by the highly sensitive ICP-MS technique.

Particularly, olive oil is considered the basis of the Mediterranean diet and simultaneously belongs among the most traded products. Consequently, the assurance of authenticity, high quality and purity of olive oil is a matter of great importance. For this purpose, investigating olive oil by means of ICP-MS analysis gains widespread scientific attention [9,126,127,128,129,130,131,132,133,134,135,136,137,138,139,140]. A considerable amount of literature has been published on Spanish, Italian, Tunisian, and Greek olive oil varieties. Preliminary work on multi-elemental analysis of olive oil was undertaken by Benincasa (2007) [126] who presented a simple and rapid ICP-MS method for the geographical origin discrimination of olive oil from various Italian regions. The final records revealed that Fe, Mg, Sr, Ca, and As led to discrimination of the samples. Systematic examination of geographical discrimination of Italian extra-virgin olive oils (EVOO) [127] and European EVOO [128] was reported in 2010 by Camin et al. The authors explored both C, H, and O isotopic ratios (IRMS) and elemental composition (ICP-MS) of olive oil samples. In case of the different European olive oils, the combination of the three isotopic ratios and the selected elements Mg, Al, K, Ca, V, Mn, Ni, Zn, Rb, Sr, Ce, Sm, Cs, La, Eu, and U showed an adequate geographical classification. Similarly, Gumus et al. [131] found that the *δ*^13^C isotope ratio and the elements Fe, Zn, Ca, Cu, and Mn were effective tracers for determining the origin of VOO from different locations of western Turkey. In a comprehensive study Aceto and co-workers [133] investigated the mineral composition of EVOO and especially the importance of lanthanides. The researchers carried out a number of experiments investigating the elemental composition of soil, olive leaves, olive seeds, olive pericarps, and olive oil *Taggiasca* variety in order to examine the effect of the production chain. The *Taggiasca* olive oils was further distinguished from EVOO of different regions. It was shown that Tm and Y were the primary discrimination elements. Similarly, Nasr et al. [9] concluded that Cu, Cr, Fe, Mn, Sr, V, and Zn facilitated to the accurately prediction of country of origin between European and Tunisian olive oils. In an attempt to certify the purity of the edible oils and to detect possible contaminations by mixing oils Llorent-Martínez et al. [139] (2011) and de Souza et al. [140] (2022) investigated the mineral composition of different types of edible oils and fats. Llorent-Martínez and co-workers observed correlations between Cr, Cu, Fe, and Mn and the different investigated oils. In the study of de Souza et al. [140], the authors achieved discrimination of the different types of oils and fat based on 9 elements: Cd, Cr, Cu, Fe, Mn, Ni, Ti, V, and Zn.

Despite the fact that olive oil is obtained from olives, there is a relatively small number of studies on elemental characterization and geographical discrimination [133,134,137,138]. In a recent study, Pucci and co-workers [138] suggested that the elements Sr, Cu, Rb, Ti, Ni, Cr, V, and Co were the most sufficient variables in the discrimination of diverse olive cultivars in Italy.

Recently, Amit and co-workers [141] identified the geographical origin of virgin coconut oil (VCO) produced in various regions. It was suggested that the combination of ICP-MS analysis with multivariate chemometrics were able to authenticate the origins of the VCO.

### 4.7. Honey

Honey is considered nature’s sweet superfood due to its beneficial properties and can find multiple applications in cooking, baking, and beverages. Because of its raw form the adulteration is easier by adding cheaper sweeteners (corn, sugar, and rice cane syrups), aiming to financial gain. Developing advanced, accurate and sensitive analytical methodologies for testing and authenticating the purity and the origin of honey is a necessity.

Literature review revealed that research has been focused on the classification of honey botanically and geographically. Carbon stable isotope ratio and elemental analysis are commonly used for the determination of the authenticity and tracing of honey [142,143,144,145,146,147,148,149,150,151]. In 2011, Chudzinska and Baralkiewicz [142] investigated the elemental composition of 140 honey samples of three types (honeydew, buckwheat, and rape) from 16 regions of Poland. They suggested Al, Mg, and Zn as best tracers for the geographical classification of samples. A key study of Zhou et al. [146], in Scientific Reports (2018), measured both C stable isotope ratio and trace elements produced in several countries worldwide. The additional sweeteners in an adulterated honey can be identified by determining the C stable isotope ratio. The sugar of these additives is produced by the C-4 metabolic pathway of plants (C-4 plants) while sugar of pure honey by C-3 metabolic pathway (C-3 plants). As a consequence, there is a disagreement between the *δ*^13^C values for C-4 (−10‰ to −20‰) and C-3 (−22‰ to −33‰) plants [152,153]. As second step, the authors further examined the mineral composition of pure honey samples in order to authenticate their origin. Generally, it was found that Ba, Ca, Fe, Mg, Mn, P, Na, and Sr exhibit variations in their concentrations according to production area of honey. Notwithstanding, further analysis and comparison of specific elements and countries led to more accurate classifications.

A comprehensive review on analytical techniques of honey authentication was published by Tsagkaris et al., in 2021 [154]. Among other analytical techniques, ICP-MS is primarily used for the determination of multi-elemental composition of honey samples from different geographical origins.

### 4.8. Cereals

Cereals, including wheat [155,156,157,158,159,160], corn (maize) [161], rice [162,163,164,165,166,167,168,169,170,171,172,173,174,175,176] and others, are the most important class of plants contributing essential nutrients and energy to human diet. Cereal production ranks among the largest in the food market due to the high demand globally. Authenticity problem is the main concern of the cereal grain trade, particularly in case of rice in which the percentage of mislabeled products is continuously increasing.

Preliminary work on geographical authenticity of wheat was performed by Branch et al., in 2002 [155]. The authors investigated the isotope analysis of Cd, Pb, Se and Sr, with the use of ICP-MS, on wheat samples from certain geographical origins. A detailed study of Podio and co-workers [157], reported the elemental and isotopic fingerprint of Argentinian wheat and correlated them with the soil and water of the certain studied regions. They demonstrated that Ba, ^87^Sr/^86^Sr, Co, Mo, Zn, Mn, Eu, *δ*^13^C, and Na were efficient variables for geographical discrimination of wheat. In a similar study, Liu et al. [159] came to the conclusion that Mn, Sr, Mo, and Cd led to correct classification of wheat samples from various regions of China. In 2017, Wang et al. [161] determined the elemental fingerprinting of maize samples by using ICP-MS. The origin of samples was certified based on the differentiations between the concentrations of the elements Na, Cr, Rb, Sr, Mo, Cs, Ba, and Pb.

**Figure 8:**
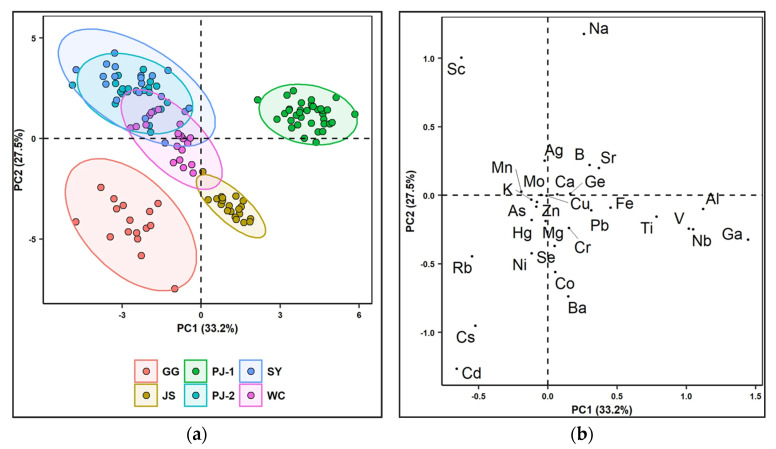
Plots with the results of PCA analysis of the measured analytes. (**a**) Resulting scoring diagram of the 1st and 2nd PCs; (**b**) Loading diagram of all analytes for the first two PCs. Figure is reprinted with permission from Ref. [172].

Respecting the rice, it was constantly being the subject of authentication studies. According to literature, there is a high number of publications about the verification of geographical origin of rice samples from all over the world. In 1999, the group of Kokot [162,163] studied the elemental composition of Vietnamese rice by combining different analytical techniques including ICP-MS, ICP-AES, and FAAS creating an element profile for the studied samples. A successful geographical discrimination was achieved by comparing the Vietnamese rice with the Australian one, based on Mn, and Mo elements. In 2002, Kelly et al. [164] investigated the C, and O stable isotope ratios and multi-elemental (B, Ho, Gd, Mg, Rb, Se, and W) analysis for the determination of the geographical origin of long grain rice, with the employment of IRMS and ICP-MS, respectively. It was suggested that B and Mg could be used as discriminative tracers. An interesting study was published by Qian et al. [170], in which the authors investigated how the fertilizers and pesticides affected the elemental composition of rice and what was the impact on the origin determination. In 2021, Xu et al. [172] developed an accurate analytical method for the authentication of the geographical origin of Chinese GI rice samples by combining ICP-MS and principal component analysis (PCAs). Figure 8a depicts the separation between the different types of GI rice; separation is clear for few samples while the rest could not be classified. In Figure 8b was shown the loading plot 1st and 2nd principal components; the authors claimed that Al, Ga, Nb, V, and Ti contributed to the first two PCAs.

The same year, Kongsri et al. [174] studied the tracing and authenticity of Thai Hom Mali rice combining stable isotope and elemental analysis. Classification of the geographical origin of samples was achieved based on Mn, Rb, Co, Mo, and *δ*^18^O.

Three extensive reviews with respect to traceability and authenticity of rice have been published by Qian et al. [177] and Maione and Barbosa [5], in 2019, and by Quinn et al. [6], in 2022.

### 4.9. Seeds and Nuts

Seeds and nuts constitute an essential part of human diet with a wide variety of products. As all the aforementioned foodstuffs, seeds and nuts are also examined for fraudulent practices possessing not only financial impact but also a high human health risk due to allergic ingredients some of them contain.

The existing literature on authentication of coffee [178,179,180,181,182,183] and cocoa beans [184,185,186] focuses mostly on the isotopic composition and multi-elemental concentrations measurements. In 2011, Rodriguez et al. [179] supported that the use of S, O, C, N, and Sr isotope ratios and elemental composition could lead to the geographical discrimination of green coffee beans originated from Hawaiian islands. In a similar work, Santato and co-workers [182] used IRMS and ICP-MS techniques to examine and classify samples of green coffee beans from different places of the world. Recently, Albals et al. [180] investigated the elemental composition by means of essential and toxic metals in green coffee beans from Brazil, Ethiopia, Kenya, Columbia, and India. As regards cocoa beans, there are relatively few authentication studies with the employment of ICP-MS technique. In 2016, Bertoldi et al. [184] determined, for the first time, the elemental profile of cocoa beans (from Africa, Asia, Central and South America) for tracing the geographical origin. The resulting records and the statistical approach, they followed, led to the selection of Ag, As, Ba, Be, Bi, Ca, Cd, Co, Cr, Cs, Cu, Fe, Ga, Hg, K, Li, Mg, Mn, Na, Ni, P, Rb, Se, Sr, Th, Tl, U, Y and Zn as efficient tracers. A similar investigation of Acierno et al. [185] revealed Fe, Cr, and Cd as potential geographical tracers.

A critical review in Food Science and Nutrition for traceability, authenticity and sustainability of cocoa beans and their derivatives was published in 2022 by Perez and co-workers [187].

Legumes are prominent members in human diet due to their nutritional value containing high protein and mineral element concentrations. They are also considered as the base of the vegetarian and vegan diet since they can substitute meat. This reason leads to a higher demand of legumes, the last decades. There are few studies on authentication and traceability of legumes [160,188,189,190,191,192] with the most studies being on fava beans. In particular, in 2014, Drivelos et al. [189] suggested the use of REE or their combination with trace elements for the geographical discrimination of the PDO “Fava Santorinis” from different Greek varieties of split peas. The results revealed that the combination of all elements (lanthanides and trace elements) provided the best geographical classification. Two years later, Drivelos and co-workers [190] examined the variations on elemental composition of PDO “Fava Santorinis” through three harvesting years. The study revealed that, in case of “Fava Santorinis”, there is discrimination of the fava beans samples from different harvesting years, while fluctuations were not observed on REEs composition throughout the years.

**Figure 9:**
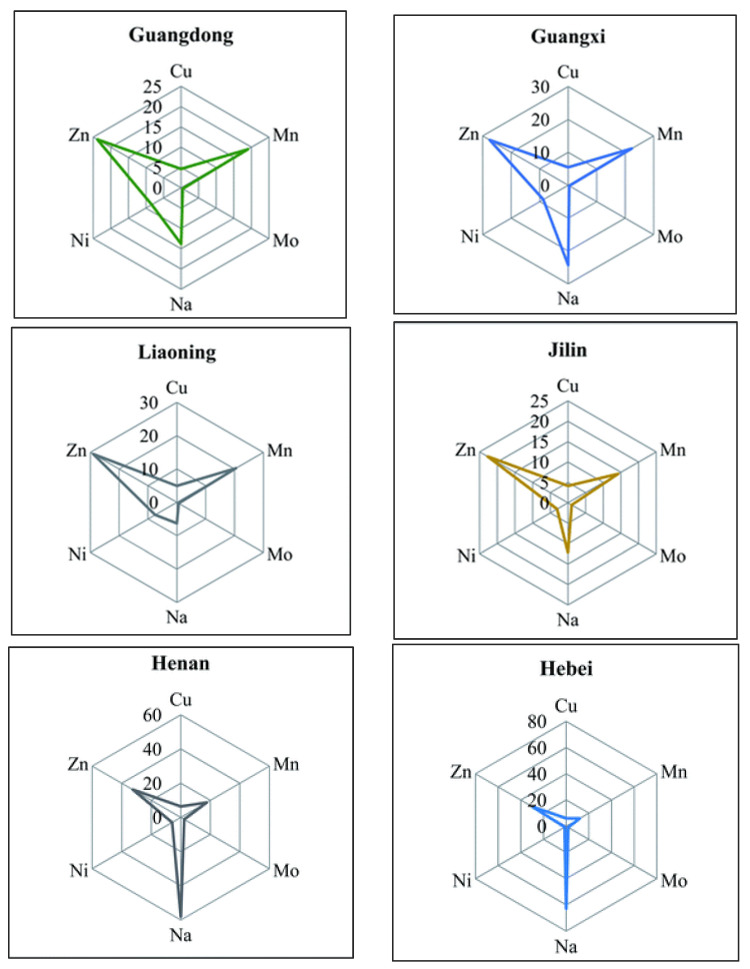
Radar plots of the elements Cu, Zn, Ni, Na, Mo and Mn in six different regions of China. Figure is reprinted with permission from Ref. [195].

Similarly, nuts (almonds, hazelnuts etc.) are well-known superfoods; they are an excellent source of protein, fiber, fats, vitamins, and minerals. Literature research showed an increasing interest in studying the elemental composition of nuts for geographical origin authentication [193,194,195,196,197,198] the last five years. The first attempt to classify hazelnuts according to their geographical origin was accomplished by Oddone et al. [193], in 2009. The authors investigated the elemental concentrations (trace elements and lanthanides) on hazelnuts and the soils from their grown region. The results confirmed the correlation of lanthanide distribution between hazelnuts and soil samples. Recently, Chen and co-workers [195] examined the concentrations of macro (K, Ca, Mg, Na, and Al), micro (Fe, Zn, Mn, Ni, Sr, Mo, Cu, Se, V, and Co), and toxic (As, Cd, Cr, and Pb) elements, with ICP-MS, in peanuts from different regions of China. Linear discriminant analysis (LDA) on all 19 elements resulted the geographical discrimination of 97% for all regions. Moreover, the authors performed radar plotting to display the elemental distribution among the different origins (Figure 9).

### 4.10. Spices and Herbs

In the spices/herbs branch fraudulent activities can occur in the form of mislabeling, and adding fillers (i.e., flour, chalk etc.) and tend to become the most vulnerable food class [199]. More recent attention has focused on the development of novel methodologies for tracking the spices/herbs supply chain. In particular, the last five years, ICP-MS has a dynamic appearance in the field of geographical origin authentication of spices and herbs [200,201,202,203,204,205,206,207,208,209,210,211]. Special attention has been paid to saffron due to its relatively high value. In 2019, D’Archivio et al. [207] examined saffron samples produced in different Italian territories. Geographical discrimination was achieved based on the analysis of the most efficient variables which were: Li, B, Na, Ga, Rb, Sr, Zr, Nb, Cs, Ba, Sm, and Hf. Perini and co-workers [206] worked on similar project by combining stable isotope ratio and multi-elemental analysis. The authors analyzed 67 saffron samples from Italy, Iran and Morocco succeeded the geographical discrimination based on the elements *δ*^13^C, *δ*^34^S, *δ*^15^N, *δ*^18^O, *δ*^2^H, K, Cr, Mn, Ni, Zn, Rb, Sr, Mo, Cs, Nd, Eu, Pb, and Ni.

An interesting review has been written, few years ago, by Galvin-King et al. [212] about herbs and spices industry. The authors clearly described the global spice and herb production chain, mentioning the consequences of adulteration on economy and public health. All the analytical methods for the safety control of spice and herbs were also referred.

Above all herbs, tea consists unique sector since it is the most popular beverage worldwide following the fresh water. A growing body of literature has focused on the tracing of tea origins with the use of ICP-MS technique [213,214,215,216,217,218,219,220,221] to further improve the accuracy of their measurements. A detailed study has been performed, in 2020, by Liu et al. [214,215], who examined the stable isotope ratios of C, N, H, O and various elements, through EA-IRMS and ICP-MS, of Chongqing tuo and Pu’er teas. In case of Chongqing tuo teas, the authors concluded to *δ*^2^H, *δ*^18^O, ^98^Mo/^95^Mo, ^96^Mo/^95^Mo, and ^98^Mo/^96^Mo as the most sufficient tracers while in Pu’er tea project the geographical discrimination was achieved based on the stable isotope ratio of *δ*D, *δ*^13^C, and ^154^Sm/^152^Sm.

Currently, a comprehensive literature review was published by Shuai et al. [222], summarizing the analytical techniques for the authentication of tea and the factors that influence the content of these measurements.

**Table 2:** Table containing the overview of the literature regarding the authenticity and traceability of agricultural products, foods and beverages.

	Product	Measured Elements	Region	References
1.	Alcoholic Beverages (Wine)	Li, Be, Na, Mg, Al, K, Ca, Sc, Ti, V, Mn, Co, Ni, Ga, As, Se, Rb, Sr, Mo, Cs, Ba, La, Ce, W, and Pb	Australia	[40]
2.	Alcoholic Beverages (Wine)	Al, As, Ba, Be, Bi, Ca, Cd, Co, Cr, Cs, Cu, Fe, Ga, In, K, Li, Mg, Mn, Ni, Pb, Rb, Se, Na, Ag, Sr, Tl, V, and U	Romania	[41]
3.	Alcoholic Beverages (Wine)	Al, Cd, Co, Cr, Cu, Li, Mn, Ni, P, Pb, Rb, Sr, and Zn	California (USA)	[42]
4.	Alcoholic Beverages (Wine)	B, Ba, Ca, Co, Cr, Cs, Cu, Fe, Ga, K, Li, Mg, Mn, Na, Ni, P, Rb, S, Sc, Si, Sr, Ti, Zn, Zr, Al, As, Cd, Ce, La, Mo, Nd, Pb, Sb, Sn, U, V, W, and Y	Spain	[43]
5.	Alcoholic Beverages (Wine)	Al, As, B, Ba, Ca, Co, Cu, Fe, K, Mg, Mn, Na, Ni, P, Pb, Sr, and Zn	Portuguese	[44]
6.	Alcoholic Beverages (Wine)	^87^Sr/^86^Sr	Romania	[61]
7.	Alcoholic Beverages (Wine)	Na, Mg, Al, K, Ca, Mn, Fe, Cu, Zn, Rb, Sr, Li, Cd, Cs, and Ba	China	[60]
8.	Alcoholic Beverages (Wine)	Ce, Dy, Er, Eu, Gd, Ho, La, Lu, Nd, Pr, Re, Sm, Ta, Tb, Tm, V, Y, and Yb	Italy	[64]
9.	Alcoholic Beverages (Wine)	As, Be, Bi, Co, Cr, Cu, K, Li, In, Tl, Se, Rb, V, U, Mg, Ni, Ba, Al, Cd, Fe, Ag, Ni and Zn	Romania	[45]
10.	Alcoholic Beverages (Wine)	Na, Mg, P, K, Ca, Cu, Co, Cr, Zn, Sn, Fe, Mn, Li, Be, B, V, Sr, Ba, Al, Ag, Ni, As, Sn, Hg, Pb, Sb, Cd, Ti, Ga, Zr, Nb, Pd, Te, La, Sm, Ho, Tm, Yb, W, Os, Au, Tl, Th, and U	Greece	[46]
11.	Alcoholic Beverages (Wine)	Li, V, Co, Ni, Ga, Mo, Cd, Sb, Cs, Ba, Ce, Nd, Ta, W, Tl, Pb, P, B, Si, Ca, Mn, Sr, K, and Rb	California (USA)	[47]
12.	Alcoholic Beverages (Wine)	Mg, K, Ca, V, Mn, Fe, Co, Ni, Cu, Zn, Ga, As, Se, Rb, Sr, Mo, Cd, Ba, Pb, and U. ^207^Pb/^206^Pb, ^208^Pb/^206^Pb, ^204^Pb/^206^Pb, and ^87^Sr/^86^Sr	Argentina	[62]
13.	Alcoholic Beverages (Wine)	Li, Be, V, Mn, Co, Ni, Cu, Ge, As, Rb, Sr, Mo, Cd, Ba, Hg, Tl, Pb, and Bi	Argentina	[48]
14.	Alcoholic Beverages (Wine)	Ag, Al, As, Ba, Be, Bi, Ca, Cd, Co, Cr, Cs, Cu, Fe, Ga, In, K, Li, Mg, Mn, Na, Ni, Pb, Rb, Se, Sr, Tl, V, U, Zn, and Hg	Romania	[63]
15.	Alcoholic Beverages (Wine)	Cu, Ni, Ca, Fe, B, Mg, As, Sb, Mn, Sn, P, Al, Zn, U, Sr, Cr, S, Co, Ba, La, Mo, Ti, Pb, Ce and V	Okanagan Valley (B.C., Canada)	[38]
16.	Alcoholic Beverages (Wine)	Li, Be, Mg, Al, P, Cl, Ca, Ti, V, Mn, Fe, Co, Ni, Cu, Zn, As, Se, Br, Rb, Sr, Mo, Ag, Cd, Sb, I, Cs, Ba, La, Ce, Tl, Pb, Bi, Th, and U	Okanagan Valley, and Niagara Region (Canada)	[49]
17.	Alcoholic Beverages (Wine)	Al, As, Ba, Be, Bi, Cd, Co, Cr, Cu, Fe, Li, Mn, Mo, Ni, Pb, Se, Sr, Ti, Tl, V, Zn, U, Sn, Sb, and Ga	Croatia	[50]
18.	Alcoholic Beverages (Wine)	Sr, Rb, Ni, Co, Pb, Mn, Cd, Ga and Cs	New Zealand	[65]
19.	Alcoholic Beverages (Wine)	Na, Mg, P, K, Ca, Al, Cr, Mn, Fe, Co, Ni, Cu, Zn, As, Se, Sr, Cd, Cs, Ba, and Pb	Spain	[51]
20.	Alcoholic Beverages (Champagne)	K, Ca, Mg, Na, B, Fe, Al, Mn, Sr, Rb, Ba, Cu, Ni, Pb, Cr, and Li	6 different brands of different vintages between 1983 and 2016	[52]
21.	Alcoholic Beverages (Wine)	Al, As, B, Ba, Ca, Ce, Cs, Co, Cr, Cu, Er, Eu, Fe, Ga, K, Mg, Mn, Mo, Na, Ni, P, Pb, Rh, Rb, Sb, Sn, Sr, Ti, Tl, Zn, and V	West coast of the USA	[53]
22.	Alcoholic Beverages (Wine)	Ag, Al, As, B, Ba, Be, Ca, Cd, Ce, Co, Cr, Cs, Cu, Dy, Er, Eu, Fe, Ga, Gd, Hf, K, La, Li, Mg, Mn, Mo, Na, Nd, Ni, P, Pb, Pr, Rb, Re, Rh, Sb, Se, Sn, Sr, Ti, Tl, Tm, U, V, W, Yb, and Zn	California (USA)	[54]
23.	Alcoholic Beverages (Wine)	Ag, B, Ba, Be, Bi, Cd, Co, Cr, Cu, Li, Mn, Mo, Ni, Pb, Rb, Sb, Sn, Sr, Te, Tl, U, and Zn	Italy, France, Poland, Spain, Slovakia, Australia, Portugal, Bulgaria, Germany, Hungary, Moldova, Chile, Austria, South Africa, New Zealand, Ukraine, Argentina, Czech Republic, Greece, UK, Armenia, and USA	[55]
24.	Alcoholic Beverages (Wine)	Li, Be, Al, Sc, V, Cr, Mn, Fe, Co, Ni, Cu, Zn, Ge, Br, Rb, Sr, Y, Zr, Nb, Pd, Ag, Cd, Ba, Pr, Nd, Sm, Eu, Gd, Tb, Dy, Ho, Er, Tm, Yb, Lu, Hf, Ta, W, Re, Pt, Tl, Pb, Bi, U, Mo, Sn, Sb, La, and Ce	Portuguese	[56]
25.	Alcoholic Beverages (Wine)	Na, K, P, Mg, and Ca	Greece	[57]
26.	Alcoholic Beverages (Wine)	La, Ce, Pr, Nd, Sm, Eu, Gd, Tb, Dy, Ho, Er, Tm and Yb	Italy	[7]
27.	Alcoholic Beverages (Wine)	Ag, Al, As, B, Ba, Bi, Cd, Co, Cr, Cu, Fe, Hg, Li, Mn, Mo, Na, Ni, Pb, Sb, Se, Sn, Sr, Ti, Tl, V, Zn, and Zr	Poland	[58]
28.	Alcoholic Beverages (Wine)	Li, Al, V, Cr, Mn, Co, Ni, Cu, Rb, Sr, Mo, Ag, Cd, Ba, Tl, Pb, Bi, U, Be, Fe, As, Se, and Zn	Poland, Hungary, Moldova, and Bulgaria	[59]
29.	Alcoholic Beverages (Wine)	La, Ce, Pr, Nd, Pm, Sm, Eu, Gd, Tb, Dy, Ho, Er, Tm, Yb, and Lu.	Italy	[66]
30.	Alcoholic Beverages (Wine)	Li, Be, A1, Sc, Ti, V, Cr, Mn, Fe, Co, Ni, Cu, Zn, Ga, Ge, As, Se, Br, Rb, Sr, Y, Zr, Nb, Mo, Ru, Rh, Pd, Ag, Cd, In, Sn, Sb Te, I, Cs, Ba, La, Ce, Pr, Nd, Sm, Eu, Gd, Tb, Dy, Ho, Er, Tm, Yb, Lu, Hf, Ta, W, Re, Os, Ir, Pt, Au, Hg, Tl, Pb, Bi, Th, and U	Spain, and England	[39]
31.	Alcoholic Beverages (Whiskey)	Al, Ti, V, Cr, Mn, Fe, Ni, Co, CU, Zn, Ga, As, Se, Rb, Sr, Zr, Mo, Nb, Ru, Rh, Pd, Ag, Cd, Sn, Sb, Te, Cs, Ba, La, Ce, Pr, Nd, Sm, Eu, Gd, Dy, Ho, Er, Tm, Yb, Lu, Hf, Ta, W, Re, Ir, Pt, Au, Tl, Pb, Th, and U	USA	[67]
32.	Alcoholic Beverages (Whiskey)	Ag, Al, As, B, Ba, Be, Bi, Ca, Cd, Co, Cr, Cu, Fe, Ga, K, Li, Mg, Mn, Mo, Na, Ni, Pb, Rb, Se, Sr, Te, Tl, U, V, and Zn	Scotland, Ireland, and USA	[68]
33.	Alcoholic Beverages (Whiskey)	Ag, Al, B, Ba, Be, Bi, Cd, Co, Cr, Cu, Li, Mn, Mo, Ni, Pb, Sb, Sn, Sr, Te, Tl, U, and V	Scotland, the USA, Ireland, Poland, Japan, the United Kingdom, India, Azerbaijan, Slovakia, Wales, and Bulgaria	[69]
34.	Alcoholic Beverages (Cider)	^87^Sr/^86^Sr, Li, Be, B, Al, Sc, Ti, V, Cr, Mn, Fe, Co, Ni, Cu, Zn, Ga, As, Se, Rb, Sr, Y, Mo, Cd, Sn, Sb, Cs, Ba, La, Ce, W, Tl, Pb, Bi, Th, and U	England, Switzerland, France, and Spain	[70]
35.	Alcoholic Beverages (Cider)	Li, Be, B, Al, Sc, Ti, V, Cr, Mn, Fe, Co, Ni, Cu, Zn, Ga, As, Se, Rb, Sr, Y, Mo, Cd, Sn, Sb, Cs, Ba, La, Ce, W, Tl, Pb, Bi, Th and U	Spain, England, France, and Switzerland	[71]
36.	Alcoholic Beverages (Beer)	V, Cr, Co, Ni, As, Se, Mo, Cd, In, Sb, Cs, Pb, Bi, and U	USA	[72]
37.	Alcoholic Beverages (Beer)	Al, As, Ba, Cd, Co, Cr, Cu, Fe, Mo, Mn, Ni, Se, Sr, Pb, and Zn	Hungary, Belgium, the Czech Republic, Germany and Austria	[73]
38.	Alcoholic Beverages (Beer)	K, Ca, Mg, Fe, Al, Mn, Zn, Na, Sr, Cu, Ti, Ba, Ni, Mo, V, Cr, Pb, Co, As, Se, Sn, Sb, U, and Cd	USA and Germany	[74]
39.	Alcoholic Beverages (Beer)	Nb, Fe, Rb, Zr, Mg, Ni, and Zn	Cavalese, and Imér	[75]
40.	Alcoholic Beverages (Liquor)	V, Cr, Mn, Ni, Co, As, Se, Sr, Mo, Cd, Sb, Ba, Pb, Bi, Al, Fe, and K	China	[76]
41.	Milk and Dairy (Cow and Buffalo Milk)	P, S, K, Ca, V, Cr, Mn, Fe, Co, Zn, Ga, Rb, Sr, Mo, Cs and Ba	Italy	[77]
42.	Milk and Dairy (Cow Milk)	Li, Al, Cr, Mn, Fe, Co, Ni, Cu, Zn, As, Se, Sr, Cd, Ba, Pb, and Bi	Sri Lanka	[8]
43.	Milk and Dairy (Cow and Goat Milk)	Al, V, Cr, Mn, Fe, Co, Ni, Cu, Zn, As, Se, Sr, Cd, Cs, Ba, Pb, U, La, Ce, Pr, Nd, Sm, Eu, Gd, Tb, Dy, Ho, Er, Yb, Lu, and Y	Italy	[78]
44.	Milk and Dairy (Cow Milk)	Sc, Ti, V, Cr, Mn, Fe, Co, Ni, Cu, Zn, Rb, Sr, Y, Zr, Sn, Sb, Ba, La, Ce, Pr, Nd, Sm, Eu, Gd, Tb, Dy, Ho, Er, Tm, Yb, Lu, Pb, Th, and U	Italy	[79]
45.	Milk and Dairy (Cheese)	Ca, Mg, Na, K, Cu, Mn, Mo, and I	Finland, England, Germany, Austria, France, and Switzerland	[80]
46.	Milk and Dairy (Cheese)	Li, Na, Mn, Fe, Cu, Se, Rb, Sr, Mo, Ba, Re, Bi, and U	Italy	[81]
47.	Meat (Yak)	Na, Mg, Al, K, Ca, Sc, V, Cr, Mn, Fe, Co, Ni, Cu, Zn, As, Se, Rb, Sr, Y, Mo, Ru, Rh, Pd, Ag, Cd, Sn, Sb, Te, Cs, Ba, La, Ce, Pr, Nd, Sm, Eu, Gd, Tb, Dy, Ho, Er, Tm, Lu, Yb, Hf, Ir, Pt, Au, Tl, Pb, Th, and U	Qinghai-Tibetan	[84]
48.	Meat (Rabbit)	As, Be, Ca, Cd, Co, Cr, Cu, Fe, Li, Mg, Mn, Mo, Ni, Pb, Sb, Se, Sr, Ti, Tl, V, Zn, Ce, Dy, Er, Eu, Gd, Ho, La, Lu, Nd, Pr, Sm, Tb, Tm, Yb, Sc, Y, Th and U	Lemnos (Greece)	[85]
49.	Meat (Pork)	Ba, Be, Bi, Cd, Co, Cr, Cu, Cs, Ga, Li, Mn, Ni, Pb, Rb, Se, Sr, U, and V	Korea, USA, Germany, Austria, Netherlands, and Belgium	[87]
50.	Meat (Mutton)	Be, Na, Al, Ca, V, Cr, Mn, Fe, Co, Ni, Cu, Zn, As, Se, Ag, Sb, Ba, Tl, Pb, Th, and U	China	[88]
51.	Meat (Cured Ham)	Zn, Fe, Rb, Cu, Sr, Al, Mn, Se, Ni, Cs, Cr, Ba, Li, As, Pb, V, Cd, Sc, Co, Ga, Tl, Y, Nd, Gd, Pr, Be, U, Sm, Dy, Yb, Eu, Ho, Tb, Tm, Rb/Sr, and ^87^Sr/^86^Sr	Europe	[89]
52.	Meat (Rabbit)	Ce, Dy, Er, Eu, Gd, Ho, La, Lu, Nd, Pr, Sc, Sm, Tb, Tm, Y, Yb, Th, and U	Lemnos (Greece)	[86]
53.	Meat (Beef)	Na, Al, K, V, Cr, Mn, Fe, Ni, Cu, Rb, Sr, Mo, Cs, and Ba	Europe, USA, South America, Australia, and New Zealand	[83]
54.	Fish and Seafood (Sea Cucumber)	Al, V, Cr, Mn, Fe, Co, Ni, Cu, Zn, As, Se, Mo, Cd, Hg, and Pb	Bohai Sea, Yellow Sea, and East China Sea (China)	[92]
55.	Fish and Seafood (Mussels)	Ag, As, Ba, Cd, Co, Cr, Cu, Ga, Mn, Mo, Nb, Ni, Pb, Rb, Sb, Se, Sn, Sr, Te, Tl, V, Zn, Ta, Zr; Ce, Dy, Er, Eu, Gd, Ho, La, Lu, Nd, Pr, Sm, Th, Tm, U, Y, and Yb	Spain, and France	[94]
56.	Fish and Seafood (Salmon)	B, Ba, Fe, K, Mg, Mn, Na, Pb, S, Sr, U and Zn	Norway	[96]
57.	Fish and Seafood (various Marine Species)	Cr, Mn, Fe, Co, Cu, Zn, As, Se, Rb, Sr, Mo, Ni, Cd, Sn, I, Ti, Ba, Hg, Pb, and Bi	China	[104]
58.	Fish and Seafood (Mussels)	Al, As, Cd, Co, Cr, Cu, Fe, Hg, Mn, Mo, Ni, Pb, Se, Sn, V, and Zn	Mediterranean Sea (Venice Lagoon)	[95]
59.	Fish and Seafood (Cuttlefish)	Na, Mg, K, Ca, P, Cu, Zn, Cr, Fe, Mo, Co, V, Ni, Mn, As, Cd, Pb, and Hg	Mediterranean Sea (Sicilian Coasts)	[97]
60.	Fish and Seafood (Manila Clam)	Na, Mg, Al, K, V, Mn, Fe, Co, Cu, Zn, As, Se, Rb, Sr, Mo, Pd, Cd, Sn, Sb, Cs, Ba, La, Ce, Pb, and U	China	[98]
61.	Fish and Seafood (Mitten Crab)	Na, Mg, Al, K, Ca, Mn, Cu, Zn, Sr, and Ba	China	[99]
62.	Fish and Seafood (Sea Bass)	La, Eu, Ho, Er, Lu, and Tb	Mediterranean Sea	[100]
63.	Fish and Seafood (Sea Cucumber)	Li, V, Cr, Mn, Co, Ni, Cu, As, Sn, Sr, Ag, Cd, Se, Ba, Pb, Bi, Y, La, Ce, Pr, Nd, Sm, Eu, Gd, Tb, Dy, Ho, Er, Tm, Yb, Lu, and Sc	China	[93]
64.	Fish and Seafood (Shrimps)	Pb, Cd, As, P, and S	Senegal, Mozambique, North Atlantic, Argentina, and Nigeria	[101]
65.	Fish and Seafood (Caviar)	Cl, Na, P, S, K, Mg, Ca, Zn, Br, Fe, Mn, Si, Sr, Rb, Cu, I, Se, As, Ba, Al, B, Co, Pb, Ag, Mo, Li, Ti, Hg, Cs, Ni, Ge, Sn, Cd, V, Cr, Sb, Pb, Te, U, Tl, Zr, Nd, Ga, Rh, La, Y, Ce, W, Be, Ta, Bi, Gd, Ru, Pr, Se, Sm, Th, Eu, Re, Dy, Au, Nb, Er, Yb, Hf, Tb, Ho, Pt, Tm, Os, Lu, and Ir	Sweeden, and Finland	[102]
66.	Fish and Seafood (Anchovy)	Li, Be, B, Al, V, Cr, Fe, Mn, Ni, Cu, Zn, Co, Ga, Ge, As, Se, Rb, Sr, Zr, Mo, Ru, Cd, In, Sn, Sb, Te, Cs, Ba, Hf, Ta, Re, Pt, Tl, Pb, Bi, Th, La, Ce, Pr, Nd, U, Y, Sm, Eu, Gd, Tb, Dy, Ho, Er, Tm, Yb, Lu, Na, Mg, P, K, Ca, Mn, Cu, Zn	Cantabria, Tunisia, and Croatia	[103]
67.	Vegetables (Onion)	Al, Ba, Ca, Cd, Ce, Cr, Dy, Eu, Fe, Ga, Gd, Ho, La, Mg, Mn, Na, Nd, Ni, Pr, Rb, Sm, Sr, Tl, Y, and Zn	Calabria (Italy)	[106]
68.	Vegetables (Onion)	Co, Ni, Cu, Rb, Mo, Cd, and Cs	Japan, China, the United States, New Zealand, Thailand, Australia, and Chile	[107]
69.	Vegetables (Garlic)	Cr, Ni, Cu, As, Se, Sb, Ba, Pb, Zn, Fe, Mg, Ca, Al, Na, K, Mn and Cd	Spain, Tunisia, and Italy	[108]
70.	Vegetables (Garlic)	Li, B, Na, Mg, P, S, Ca, Ti, Mn, Fe, Cu, Ni, Zn, Rb, Sr, Mo, Cd, and Ba	Argentine, Canada, Chile, Korea, Mexico, Pakistan, Thailand, United States, and Vietnam	[109]
71.	Vegetables (Soybean)	Ag, As, Ba, Ca, Cd, Co, Cr, Cu, Fe, K, Mg, Mn, Mo, Na, Ni, Pb, Sb, Se, Sn, Sr, Ti, Tl, V and Zn	Zhejiang, Heilongjiang, Hebei, Inner Mongolia, Henan, Hainan, and Fujian (China)	[110]
72.	Vegetables (Tomato, Pepper, Eggplant)	Mn, Fe, Cu, Zn, Cr, Ni, Cd, and Pb	Romania	[111]
73.	Vegetables (Peper Capsicum annuum L.)	Ar, Ba, Ca, Cd, Co, Cr, Cs, Cu, Fe, K, Mn, Mg, P, Mo, Ni, Na, Pb, Rb, Sb, Sn, Tl, Y, Sr, and Zn	Xiazi, Huaxi, and Hezhang (China)	[112]
74.	Vegetables (Chili peppers)	Al, As, Ba, Ca, Cd, Ce, Co, Cr, Cs, Cu, Dy, Fe, Ga, La, Li, Mg, Mn, Na, Nd, Ni, Pb, Pr, Rb, Sc, Se, Sr, Tl, Tm, V, Y, Yb, and Zn	Calabria (Italy)	[113]
75.	Vegetables (Tomato)	Al, As, Ba, Be, Ca, Cd, Ce, Cu, Dy, Fe, K, La, Lu, Mg, Mn, Na, Nd, Pb, Rb, Sm, Sr, Th, U, V, and Zn	Italy, China, Greece and California	[114]
76.	Vegetables (Tomato)	Y, La, Ce, Pr, Nd, Sm, Eu, Gd, Tb, Dy, Ho, and Yb	Italy	[115]
77.	Vegetables (Tomato)	Li, Be, B, Na, Mg, Al, P, K, Ca, V, Cr, Mn, Fe, Co, Ni, Cu, Zn, Ga, Ge, As, Se, Rb, Sr, Y, Mo, Ag, Cd, Sn, Sb, Cs, Ba, La, Ce, Pr, Nd, Sm, Eu, Gd, Dy, Ho, Tm, Yb, Ir, Tl, Pb, U	Italy	[116]
78.	Vegetables (Chinese cabbage)	Mn, Cu, Sr, Ba, S, Co, Cr, Li, Ni, Ti, V, and Zn	China, and Korea	[117]
79.	Mushrooms (Truffles)	Li, Na, Mg, Al, K, V, Cr, Mn, Co, Ni, Cu, Ga, Rb, Sr, Mo, Ag, Cd, Te, Ba, Tl, Pb, Bi, U, Sc, Y, La, Ce, Pr, Nd, Sm, Eu, Gd, Tb, Dy, Ho, Er, Tm, Yb, Lu, Th, Be, B, Fe, Zn, As, and Se	Bulgaria, Romania, Croatia, Hungary, Iran, Slovenia, Italy, Spain, Australia, and China	[118]
80.	Fruits (Lemon)	K, Ca, Mg, Na, Fe, Zn, B, Cu, Al, Mn, Ni, Cr, Pb, Co, As, Se, Cd, Sb, V, La, Ce, Pr, Nd, Sm, Eu, Gd, Tb, Dy, Ho, Er, Tm, and Lu	Italy, and Turkey	[119]
81.	Fruits (Apples)	Ag, Al, As, Be, Bi, Cd, Co, Cr, Cu, Fe, Ga, In, Mn, Mo, Ni, Pb, Rb, Se, Sn, Tl, U, V, Zn, La, Ce, Pr, Nd, Sm, Eu, Gd, Tb, Dy, Ho, Er, Tm, and and Lu	Italy	[120]
82.	Fruits (Mango)	Li, Be, B, Na, Mg, Al, P, K, Ca, V, Cr, Mn, Fe, Co, Ni, Cu, Zn, Ga, Ge, As, Se, Rb, Sr, Y, Mo, Pd, Ag, Cd, In, Sn, Sb, Te, Cs, Ba, La, Ce, Pr, Nd, Sm, Eu, Gd, Dy, Er, Tm, Yb, Re, Ir, Pt, Au, Hg, Pb, Th and U	Spain, Senegal, Ivory Coast, Equatorial Guinea, Peru, Mexico, and Brazil	[121]
83.	Fruits (Avocado)	Li, Be, B, Na, Mg, Al, P, K, Ca, Cr, Mn, Fe, Co, Ni, Cu, Zn, Ga, Ge, As, Se, Rb, Sr, Y, Mo, Pd, Ag, Cd, Sn, Sb, Cs, Ba, La, Ce, Pr, Nd, Sm, Eu, Gd, Dy, Ho, Er, Tm, Yb, Hg, Pb, and U	Spain, Brazil, Chile, Colombia, Kenya, Mexico, Peru, and South Africa	[122]
84.	Fruits (Pear)	Al, As, B, Be, Ca, Cd, Co, Cr, Cu, Fe, K, Li, Mg, Mn, Mo, Na, Ni, P, Pb, Se, Sn, Sr, Tl and Zn	Portuguese	[123]
85.	Fruits (Jackfruits)	Ba, Al, Ca, Co, Cs, Cr, Cu, Fe, Ga, S, K, Li, Mg, Mn, Na, Ni, Mo, Rb, Ti, U, B, Zn, Si, and Xe	India	[124]
86.	Fruits (Clementine)	Ag, Al, As, Ba, Be, Bi, Ca, Cd, Co, Cr, Cs, Cu, Fe, Ga, In, K, Li, Mg, Mn, Na, Ni, Pb, Rb, Se, Sr, Tl, V, U, Zn, Ce, Dy, Er, Eu, Gd, Ho, La, Lu, Nd, Pr, Sm, Sc, Tb, Th, Tm, Y, and Yb	Calabria (Italy)	[125]
87.	Oil (EVOO)	Be, Mg, Ca, Sc, Cr, Mn, Fe, Co, Ni, As, Se, Sr, Y, Cd, Sb, Sm, Eu, and Gd	Italy	[126]
88.	Oil (EVOO)	Li, B, Na, Mg, K, Ca, Mn, Co, Cu, Ga, Se, Rb, Sr, Mo, Cd, Cs, Ba, La, Ce, Nd, Sm, Eu, Yb, Tl, Pb, and U	Italy	[127]
89.	Oil (EVOO)	Mg, K, Ca, V, Mn, Zn, Rb, Sr, Cs, La, Ce, Sm, Eu, U	European Region	[128]
90.	Oil (VOO)	Y, La, Ce, Pr, Nd, Sm, Gd, Tb, Dy, Ho, Er, Tm, Yb, and Th	Zakynthos, Iraklio, Lakonia, and Messinia (Greece)	[129]
91.	Oil (VOO)	Al, As Ba, Ca, Co, Cr, Cs, Cu, Fe, Ga, Hf, K, Li, Mo, Mn, Mg Na, Sr, Nb, Ni, Pb, Rb, Sc, Se, Sn, and Ta	Spain	[130]
92.	Oil (VOO)	V, Mn, Ni, Cu, Ba, Na, K, Ca, Fe, Mg, Pb, As, Co, Cr, and Zn	İzmir, Manisa, Aydın, Muğla, Bursa, and Edremit Bay (Turkey)	[131]
93.	Oil (EVOO)	Na, Mg, V, Fe, Mn, Zn, As, Rb, Sr, Ba, and Pb	Tunisia	[132]
94.	Oil (EVOO)	Sc, Ti, V, Cr, Mn, Fe, Co, Ni, Cu, Rb, Sr, Y, Cd, Sb, Ba, La, Ce, Pr, Nd, Sm, Eu, Gd, Tb, Dy, Ho, Er, Tm, Yb, Lu, W, Tl, Pb, Th, and U	Italy (different varieties)	[133]
95.	Oil (EVOO)	B, Na, P, Ca, Li, Mg, Fe, Cu, and As	Tunisia	[134]
96.	Oil (EVOO)	Li, Be, B, Na, Mg, Al, Si, P, K, Ca, Ti, V, Cr, Mn, Fe, Co, Ni, Cu, Zn, Ga, As, Se, Rb, Sr, Zr, Nb, Mo, Ag, Cd, Sn, Sb, Te, Cs, Ba, La, Ce, Pr, Nd, Tb, Dy, W, Tl, Pb, Bi, and U	Tuscany, Umbria, Apulia, Sardinia, Sicily, Abruzzo, Campania, and Marche (Italy)	[135]
97.	Oil (EVOO)	Ag, Al, As, B, Ba, Ca, Cd, Co, Cr, Cs, Cu, Fe, Ga, Ge, K, Li, Mg, Mn, Mo, Na, Ni, P, Pb, Rb, Sb, Se, Si, Sn, Sr, Ti, V, W, Zn, Zr, Ce, Dy, Er, Eu, Gd, Ho, La, Lu, Nd, Pr, Sm, Sc, Tb, Tm, Yb, and Y	Tunisia	[136]
98.	Oil (VOO) and Olives	Al, As, Cd, Co, Cr, Cu, Fe, Ni, Pb, Sb, and V	Spain	[137]
99.	Olives	Sr, Cu, Rb, Ti, Ni, Sn, Cr, V, Co, Sb Cd, Pb, As, and Zr	Italy	[138]
100.	Oil (EVOO)	As, Ba, Ca, Cd, Co, Cr, Cu, Fe, K, Mg, Mn, Ni, Rb, Sr, Pb, V, and Zn	Tunisia, and Europe	[9]
101.	Different types of oil	Ag, As, Ba, Be, Cd, Co, Cu, Fe, Hg, Mn, Mo, Ni, Pb, Sb, Ti, Tl, and V	Spain	[139]
102.	Oil (VCO)	Na, Mg, Al, P, Ca, Cr, Mn, Fe, Ni, Cu, Zn, Se, Rb, Sr, Mo, Cs, and Pb	Kerala, Karnataka, Andhra, Tamil Nadu, Goa (India)	[141]
103.	Honey	Al, B, Ba, Ca, Cd, Cr, Cu, K, Mg, Mn, Na, Ni, Pb, Sr, and Zn	Poland	[142]
104.	Honey	Al, Cu, Pb, Zn, Mn, Cd, Tl, Co, Ni, Rb, Ba, Be, Bi, U, V, Fe, Pt, Pd, Te, Hf, Mo, Sn, Sb, P, La, Mg, I, Sm, Tb, Dy, Sd, Th, Pr, Nd, Tm, Yb, Lu, Gd, Ho, Er, Ce, and Cr	Brazil	[143]
105.	Honey	Na, Mg, P, K, Ca, Mn, Fe, Cu, Zn, Rb, Sr, and Ba	China	[144]
106.	Honey	Mn, Cu, Cr, Ni, Se, Pb, Cd, and As	Sicily, and Calabria (Italy)	[145]
107.	Honey	As, Cd, Cr, Cu, Hg, Fe, Mn, Ni, Pb, and Zn	Romania	[147]
108.	Honey	Ag, Al, As, Au, B, Ba, Be, Bi, Ca, Cd, Ce, Cs, Cr, Co, Cu, Dy, Er, Eu, Fe, Ga, Gd, Ge, Hg, Hf, Ho, Rb, K, La, Li, Lu, Mg, Mn, Mo, Na, Nb, Nd, Ni, Os, P, Pb, Pd, Pt, Pr, Re, Ru, Se, Sb, Sr, Sm, Sn, Ta, Tb, Te, Th, Tl, Tm, Ti, U, V, W, Y, Yb, Zn and Zr.	Africa, Asia, Europe, North America, and Oceania	[146]
109.	Honey	Al, As, Ba, Ca, Cd, Co, Cr, Cu, Mg, Mn, Na, Ni, K, Pb, Sr, Ti, V and Zn	Romania	[148]
110.	Honey	Al, As, Au, Ba, Co, Cr, Cs, Cu, Fe, In, Ir, Mg, Mn, Pb, Pd, Pt, Rb, Sb, Se, Te, Th, Tl, U, V, Zn, Ce, Dy, Eu, Er, Gd, Ho, La, Lu, Nd, Pr, Sm, Tb, Tm, Yb, and Zn	Santa Catarina, Paraná, and Rio Grande do Sul (Brazil)	[149]
111.	Honey	Li, Mg, Mn, Ni, Co, Cu, Sr, Ba, Pb, Y, La, Ce, Pr, Nd, Sm, Eu, Gd, Tb, Dy, Ho, Er, Tm, Yb, and Lu	Greece, Bulgaria, Romania, Italy, Thailand, and Poland	[150]
112.	Honey	Ag, As, Ba, Be, Bi, Cd, Co, Cr, Cu, Fe, Hg, Li, Mn, Mo, Ni, Pb, Sb, Sn, Sr, Te, Tl, V, and Zn	Sardinia (Italy)	[151]
113.	Cereals (Wheat)	Cd, Pb, Se, and Sr	USA, Canada, and Europe	[155]
114.	Cereals (Wheat)	Be, Na, Mg, Al, K, Ca, V, Mn, Fe, Cu, Zn, Mo, Cd, Ba, and Th)	China	[156]
115.	Cereals (Wheat)	K/Rb, Ca/Sr, Ba, ^87^Sr/^86^Sr, Co, Mo, Zn, Mn, Eu, δ13C, and Na	Buenos Aires, Córdoba, and Entre Ríos	[157]
116.	Cereals (Wheat)	Be, Na, Mg, Ca, Sc, Ti, V, Cr, Mn, Fe, Co, Cu, Zn, Ga, Se, Rb, Sr, Y, Zr, Cd, Cs, and Pb	China	[158]
117.	Cereals (Wheat)	Mg, Al, Ca, Mn, Fe, Cu, Zn, As, Sr, Mo, Cd, Ba, and Pb	China	[159]
118.	Cereals (Maize)	B, Na, Mg, Al, P, K, Ca, V, Cr, Mn, Fe, Co, Ni, Cu, Zn, As, Se, Rb, Sr, Mo, Cd, Cs, Ba, Pb, and U	China	[161]
119.	Cereals (Wheat, Barley), and Legumes (Faba Bean)	Li, Be, B, Na, Mg, Al, Si, P, S, Cl, K, Ca, Sc, Ti, V, Cr, Mn, Fe, Co, Ni, Cu, Zn, Ga, Ge, As, Se, Br, Rb, Sr, Y, Zr, Nb, Mo, Ru, Rh, Pd, Ag, Cd, In, Sn, Sb, Te, I, Cs, Ba, La, Ce, Pr, Nd, Sm, Eu, Gd, Tb, Dy, Ho, Er, Tm, Yb, Lu, Hf, Ta, W, Re, Os, Ir, Pt, Au, Hg, Tl, Pb, Bi, Th, and U	Zealand, Central Jutland, and South Jutland	[160]
120.	Rice	Ni, Mo, As, and Cd	Vietnam	[162]
121.	Rice	Ni, Mo, As, and Cd	Vietnam	[163]
122.	Rice	B, Ho, Gd, Mg, Rb, Se, and W	India, Pakistan, USA, France, Italy, Spain,	[164]
123.	Rice	Al, Fe, Co, Ni, Cu, Rb, Sr, and Ba	Japan, USA, China, and Thailand	[165]
124.	Rice	Mg, K, Ca, Na, Be, Mn, Ni, Cu, Cd, Fe, Al, Cr, Zn, Sb, and Pb	China	[166]
125.	Rice	B, Co, Sr, Mo, Cd, Cs, Ba, Pb, Ti, V, As, Se, Mn, Cu, Rb, Mg, Al, Cr, Fe, Ni, and Zn	Thailand, France, Japan, India, Italy, and Pakistan	[167]
126.	Rice	As, B, Ba, Ca, Cd, Ce, Co, Cr, Cu, Fe, K, La, Mg, Mn, Mo, P, Pb, Rb, Se and Zn	Brazil	[168]
127.	Rice	B, Mg, Al, Ti, V, Cr, Mn, Fe, Ni, Zn, Ga, As, Sr, Cd, Sn, Sb, Ba, Pb, Bi, and Tl	China	[169]
128.	Rice	Al, As, Ba, Bi, Cd, Ca, Cr, Co, Cu, Fe, Pb, Li, Mg, Mn, Mo, Ni, K, Se, Na, Sr, Tl, Ti, Zn, La, Ce, Pr, Nd, Sm, Eu, Ho, Er and Yb	China	[170]
129.	Rice	^87^Sr/^86^Sr, ^207^Pb/^206^Pb, ^208^Pb/^207^Pb, Li, Be, Na, Mg, Al, Ca, Ti, V, Cr, Mn, Fe, Co, Ni, Cu, Zn, Rb, Sr, Mo, Ag, Cd, Sn, Sb, Ba, Pb, and Bi	China, Thailand, and Malaysia	[171]
130.	Rice	B, Na, Mg, Al, K, Ca, Sc, Ti, V, Cr, Mn, Fe, Co, Ni, Cu, Zn, Ga, Ge, As, Se, Rb, Sr, Nb, Mo, Ag, Cd, Cs, Ba, Hg, and Pb	Heilongjiang, Liaoning, Jiangsu, Hubei, and Guangxi	[172]
131.	Rice	Na, Mg, Al, Si, P, S, K, Ca, Sc, Ti, Cr, Mn, Fe, Co, Ni, Cu, Zn, Ga, Ge, As, Se, Br, Rb, Sr, Y, Mo, Ba, La, Ce, Au, and Pb	India, and Pakistan	[173]
132.	Rice	Mn, Rb, Co, and Mo	Thailand	[174]
133.	Rice	B, Na, Mg, Al, K, Ca, Sc, Ti, V, Cr, Mn, Fe, Co, Ni, Cu, Zn, Ga, Ge, As, Se, Rb, Sr, Nb, Mo, Ag, Cd, Cs, Ba, Hg, and Pb	China, India, Vietnam, and Ghana	[175]
134.	Rice	Ag, Al, As, Ba, Be, Bi, Ca, Cd, Co, Cr, Cs, Cu, Fe, Ga, In, K, Li, Mg, Mn, Na, Ni, Pb, Rb, Se, Sr, Tl, U, V, Zn, Ce, Dy, Er, Eu, Gd, Ho, La, Lu, Nd, Pr, Sc, Sm, Tb, Th, Tm, and Y	Vietnam	[176]
135.	Coffee	Ba, Ca, Cu, Fe, Mg, Mn, P, Si, K, and S	Ethiopia	[178]
136.	Coffee	B, Na, Mg, Al, Cr, Mn, Fe, Co, Ni, Cu, Zn, Ga, Rb, Sr, Mo, Ba, Pb, Bi, Y, La, Ce, Pr, Sm, Nd, Eu, Dy, Th, Sc, Ho, and Gd	Hawaii, Kauai, Maui, Molokai, and Oahu	[179]
137.	Coffee	K, Mg, Ca, Fe, Al, Mn, Cu, Ba, Sr, Zn, Cr, Pb, Ni, V, Co, Ga, U, Cd, Ag, Li, In, Bi, Th, and Tl	Brazil, Ethiopia, Kenya, Columbia, and India	[180]
138.	Coffee	Na, Ca, K, Mg, Al, As, Pb, Ni, Zn, Cu, Mn, Cd, Fe, Hg, and Cr	Germany, Netherlands, Italy, Austria, Slovenia, France, Romania, Brazil, and Greece	[181]
139.	Coffee	Li, Be, B, Na, Mg, Al, P, K, Ca, Ti, V, Cr, Mn, Fe, Co, Ni, Cu, Zn, Ga, Ge, As, Se, Rb, Sr, Y, Mo, Pd, Ag, Cd, Sn, Sb, Te, Cs, Ba, La, Ce, Pr, Nd, Sm, Eu, Gd, Dy, Er, Tm, Yb, Re, Ir, Pt, Au, Hg, Tl, Pb, Bi and U	Central/South America, Africa, and Asia	[182]
140.	Coffee	Ba, Ca, Cu, Fe, K, Mg, Mn, P, Sr, Sc, and Zn	Espirito Santo, Minas Gerais, and São Paolo (Brazil)	[183]
141.	Cocoa	Li, Be, B, Na, Mg, Al, P, K, Ca, Ti, V, Cr, Mn, Fe, Co, Ni, Cu, Zn, Ga, Ge, As, Se, Rb, Sr, Y, Mo, Pd, Ag, Cd, In, Sn, Sb, Te, Cs, Ba, La, Ce, Pr, Nd, Sm, Eu, Gd, Dy, Er, Tm, Yb, Re, Ir, Pt, Au, Hg, Tl, Pb, Bi, Th, and U	Africa, Asia, Central and South America	[184]
142.	Cocoa	Na, Cr, La, Ce, Mo, Cs, Ga, Ti, Y, Ba, Rb, Zn, Sr, Fe, Mg, Al, Co, Cu, Cd, Mn, Ni, As, Pb, and V	Congo, Mexico, Ecuador, Venezuela, Costa Rica, Vanuatu, and Trinidad	[185]
143.	Cocoa	B, Ba, Ca, Cd, Co, Cr, Cs, Cu, K, Ni, Mg, Mn, Mo, P, S, Sr, V, and Zn	Africa, Asia Pasific, Central/South America	[186]
144.	Legumes (Cowpeas)	Ag, As, Ba, Be, Cd, Cs, Co, Cr, Cu, Mo, Ni, Pb, Sb, Se, Sn, Sr, Tl, Rb, V, and Zn	Argentina	[188]
145.	Legumes (Yellow Split Pea)	Y, La, Ce, Pr, Nd, Sm, Eu, Gd, Dy, Ho, Er, Yb, Th, Sc, B, Al, Cr, Mn, Fe, Co, Ni, Cu, Zn, Se, Cd, Ba, and Tl	Santorini, different places of Greece, India, Canada, USA, Iran, and Australia	[189]
146.	Legumes (Faba Beans)	Y, La, Ce, Pr, Nd, Sm, Eu, Gd, Tb, Dy, Ho, Er, Tm, Yb, Lu, Be, Al, Cr, Mn, Fe, Co, Ni, Cu, Zn, Cd, and Ba	Santorini (Greece)	[190]
147.	Legumes (Faba Beans)	Li, B, Na, Mg, Al, P, S, K, Ca, Cr, Mn, Fe, Co, Ni, Cu, Zn, As, Se, Mo, Cd, Ba, La, Hg, and Pb	Manitoba and Saskatchewan (Canada)	[191]
148.	Legumes (Common Beans)	N, Mg, P, S, K, Ca, Mn, Fe, Cu, Na, Cr, Co, Zn, and Mo	Slovenia	[192]
149.	Nuts (Hazelnuts)	La, Ce, Pr, Nd, Sm, Eu, Gd, Tb, Dy, 165Ho, Er, Tm, Yb, and Lu	Italy, and Turkey	[193]
150.	Nuts (Pistachio)	La, Ce, Pr, Nd, Sm, Eu, Gd, Tb, Dy, Ho, Er, Tm, and Yb	Greece, and Turkey	[194]
151.	Nuts (Peanuts)	K, Ca, Mg, Na, Al, Fe, Zn, Mn, Ni, Sr, Mo, Cu, Se, V, Co, As, Cd, Cr, and Pb	China	[195]
152.	Nuts (Walnuts)	Li, Be, B, Na, Mg, Al, K, Ca, Sc, V, Cr, Mn, Fe, Co, Ni, Cu, Zn, Ga, As, Se, Rb, Sr, Y, Mo, Ag, Cd, Te, Ba, La, Ce, Pr, Nd, Sm, Eu, Gd, Tb, Dy, Ho, Er, Tm, Yb, Lu, Tl, Pb, Bi, Th, and U	Switzerland, Chile, China, Germany, France, Hungary, Italy, Pakistan, Turkey, and USA	[196]
153.	Nuts (Almonds)	Li, B, Al, Ti, Mn, Fe, Ni, Cu, Zn, Rb, Y, Ag, Cd, Ba, Ce, Tl, and U	Australia, Spain, Iran, Italy, Morocco, USA	[197]
154.	Sesame seeds	Mg, Al, K, Ca, Cr, Mn, Co, Ni, Cu, Zn, Rb, Sr, Cd, Ba, and Pb	Korean, Chinese, and Indian	[198]
155.	Spices	Cr, Co, Ni, Cu, Hg, Cd, Pb, and As	17 Different Countries	[200]
156.	Spices (red pepper flake)	Al, V, Cr, Mn, Fe, Ni, Cu, Zn, As, Cd, and Pb	Southeast Anatolia Region, the Mediterranean Region, and the Central Anatolia Region (Turkey)	[201]
157.	Spices (Black pepper)	Mg, K, Ca, Ti, Cr, Mn, Fe, Co, Ni, Cu, Se, Sr, Y, Mo, Sb, Ba, Pt, and Pb	Vietnam, Pakistan, and India	[202]
158.	Spices (Chili)	Ba, Be, Co, Cr, Cu, Fe, Ga, Li, Mn, Ni, Rb, Se, Sr, V, Zn, As, Cd, In, Pb, and Tl	South Korea, China, and Vietnam	[203]
159.	Spices (Hot/Sweet Paprika)	K, Mg, Fe, Zn, Cu, Mn, B, Al, Co, Ni, Se, Mo, As, Pb, and Cd	Serbia, and Hungry (Comparison with literature samples from Spain, Turkey, and Poland)	[204]
160.	Spices	^87^Sr/^86^Sr, Rb, Sr, Y, Zr, Mo, Cd, Ba, Pb, Th, U, Mg, Ca, Sc, Ti, Cr, Mn, Fe, Co, Ni, Cu, Zn, As, and REE	Hungary, Spain, Romania, France, Senegal, China, and Germany	[205]
161.	Saffron	Li, Be, B, Na, Mg, Al, K, Ca, Sc, Ti, V, Cr, Mn, Fe, Co, Ni, Cu, Zn, Ga, As, Rb, Sr, Y, Zr, Nb, Mo, Ag, Cd, Cs, Ba, La, Ce, Pr, Nd, Sm, Eu, Dy, Ho, Er, Hf, Re, Pb	Italy, Morocco and Iran	[206]
162.	Saffron	Fe, Ca, Na, Mg, Sr, Ag, Al, As, Ba, Be, Cd, Co, Cr, Cu, Mn, Mo, Ni, Pb, Sb, Tl, V, Zn, and U	Italy, and Iran	[207]
163.	Herbs	Ag, Al, As, B, Ba, Be, Bi, Ca, Cd, Co, Cr, Cu, Fe, K, Li, Mg, Mn, Mo, Na, Ni, P, Pb, Sb, Se, Si, Sn, Sr, Ti, V, Zn, Zr	China	[208]
164.	Spices, and Herbs	Fe, Zn, Cr, Ni, Cu, Se, Pb, As, K, Ca, Mg, Na, Co, Mn, Hg and Cd	Italy, and Tunisia	[209]
165.	Spices, and Herbs	Na, Mg, K, Ca, Cr, Mn, Fe, Co, Ni, Cu, Zn, As, Se, Cd, Hg, and Pb	Algeria	[210]
166.	Herbs	Zn, Cd, Co, Cr, Cu, Ca, Mg, Mn, Mo, Ni, Pb, Sr, Fe, Na, and K	China	[211]
167.	Tea	La, Ce, Pr, Nd, Sm, Eu, Gd, Tb, Dy, Ho, Er, Tm, Yb, Lu, Sc, and Y	China	[213]
168.	Tea	^109^Ag/^107^Ag, ^138^Ba/^137^Ba, ^81^Br/^79^Br, ^112^Cd/^111^Cd, ^114^Cd/^112^Cd, ^114^Cd/^111^Cd, ^53^Cr/^52^Cr, ^72^Ge/^70^Ge, ^74^Ge/^72^Ge, ^74^Ge/^70^Ge, ^202^Hg/^200^Hg, ^7^Li/^6^Li, ^96^Mo/^95^Mo, ^98^Mo/^96^Mo, ^98^Mo/^95^Mo, ^60^Ni/^58^Ni, ^207^Pb/^206^Pb, ^208^Pb/^207^Pb, ^208^Pb/^206^Pb, ^123^Sb/^121^Sb, ^80^Se/^78^Se, ^120^Sn/^118^Sn, ^88^Sr/^86^Sr, ^47^Ti/^46^Ti, and ^48^Ti/^47^Ti, ^48^Ti/^46^Ti, ^205^Tl/^203^Tl, ^66^Zn/^64^Zn, ^68^Zn/^66^Zn, ^68^Zn/^64^Zn, ^71^Ga/^69^Ga, ^153^Eu/^151^Eu, ^154^Sm/^152^Sm, ^158^Gd/^156^Gd, ^160^Gd/^158^Gd, ^160^Gd/^156^Gd, ^164^Dy/^162^Dy, ^168^Er/^166^Er, ^174^Yb/^172^Yb, ^176^Lu/^175^Lu	China	[214]
169.	Tea	^109^Ag/^107^Ag, ^138^Ba/^137^Ba, ^81^Br/^79^Br, ^112^Cd/^111^Cd, ^114^Cd/^112^Cd, ^114^Cd/^111^Cd, ^53^Cr/^52^Cr, ^72^Ge/^70^Ge, ^74^Ge/^72^Ge, ^74^Ge/^70^Ge, ^202^Hg/^200^Hg, ^7^Li/^6^Li, ^96^Mo/^95^Mo, ^98^Mo/^96^Mo, ^98^Mo/^95^Mo, ^60^Ni/^58^Ni, ^207^Pb/^206^Pb, ^208^Pb/^207^Pb, ^208^Pb/^206^Pb, ^123^Sb/^121^Sb, ^80^Se/^78^Se, ^120^Sn/^118^Sn, ^88^Sr/^86^Sr, ^47^Ti/^46^Ti, and ^48^Ti/^47^Ti, ^48^Ti/^46^Ti, ^205^Tl/^203^Tl, ^66^Zn/^64^Zn, ^68^Zn/^66^Zn, ^68^Zn/^64^Zn, ^71^Ga/^69^Ga, ^153^Eu/^151^Eu, ^154^Sm/^152^Sm, ^158^Gd/^156^Gd, ^160^Gd/^158^Gd, ^160^Gd/^156^Gd, ^164^Dy/^162^Dy, ^168^Er/^166^Er, ^174^Yb/^172^Yb, ^176^Lu/^175^Lu	China	[215]
170.	Tea	V, Cr, Co, Ga, Sr, Mo, Cd, Pb, Na, Al, Fe, Ni, Cu, Zn, Rb, and Ba	China	[216]
171.	Tea	86 Mineral elements	China	[217]
172.	Tea	Ag, As, Ba, Be, Bi, Br, Cd, Co, Cr, Ge, Hg, Li, Mo, Nb, Ni, Pb, Rb, Sb, Se, Sn, Sr, Ti, Tl, V, Zn, Cs, Hf, Y, La, Ce, Pr, Nd, Eu, Sm, Gd, Dy, Ho, Er, Tm, Yb, Lu, Sc, and Tb	China	[221]
173.	Tea	Ag, Al, As, Ba, Be, Bi, Ca, Cd, Co, Cr, Cs, Cu, Fe, Ga, In, K, Li, Mg, Mn, Na, Ni, Pb, Rb, Se, Sr, Tl, U, V, Zn, Ce, Dy, Er, Eu, Gd, Ho, La, Lu, Nd, Pr, Sc, Sm, Tb, Th, Tm, and Y	China	[218]
174.	Tea	Ti, Cr, Co, Ni, Cu, Zn, Rb, Cd, Cs, Ba, Sr, Ca, Mg, and Mn	China	[219]
175.	Tea	Al, Ba, Ca, Cd, Ce, Co, Cr, Cs, Cu, Fe, K, Li, Mn, Ni, P, Pb, Rb, Sr, Tl, U, Y, and Zn	China	[220]

## 5. Conclusions

The present systematic review summarizes the research and development on inductively coupled plasma mass spectrometry (ICP-MS) in geographical origin authentication of agricultural products, food, and beverages. In addition to multi-elemental analysis, C, H, O, S, and Sr stable isotope ratio analysis is often utilized complementary, providing a more complete data source for confirming the research objective. The reader is able to understand the fundamentals of the ICP-MS technique in a brief tutorial presentation in Section 2, while Section 3 offers a complete overview of the research about ICP-MS in authenticity of geographical origin of food. Notwithstanding the fact that, when origin authentication and traceability of agri-food products is the main issue, ICP-MS has been the first option, confirming the superiority of the technique in the field. This is, also, confirmed by the huge number of publications on the topic and even more by the representative works of Zhou et al. [146] and Quinn et al. [6], who successfully determined the geographical origin of global honeys and Asian rice samples, respectively. Hence, it can be safely concluded that ICP-MS analysis is mature with the highest possible accuracy and precision in multi-elemental measurements with low uncertainty. Novel instrumental developments allow researchers to avoid and/or correct possible drawbacks of the technique including the interferences (spectroscopic and non-spectroscopic), Plasma Effects, Space-Charge Effects and Sample Introduction Effects which result in false data. Edible products from diverse areas have been shown to differentiate on their matrix compositions which affect the inorganic components of food substances. Exploiting this, ICP-MS is highly beneficial providing information about the geographical origin of food products due to the variety of matrices and consequently the inorganic components.

## Figures and Tables

**Table 1:** Selected typical spectroscopic interferences.

**Isotope**	**Isobaric Interference**
^40^K	^40^Ca
^50^V	^50^Cr
^64^Ni	^64^Zn
^94^Zr	^94^Mo
^113^In	^113^Cd
**Isotope**	**Double Charge Ion**
^44^Ca	^88^Sr^2+^
^69^Ga	^138^Ba^2+^
^70^Ge	^140^Ce^2+^
^85^Rb	^170^Er^2+^
^119^Sn	^238^U^2+^
**Isotope**	**Polyatomic Ions**
^28^Si	^14^N_2_^+^, ^12^C^16^O^+^
^31^P	^14^N^16^O^1^H^+^
^44^Ca	^12^C^16^O_2_^+^
^36^Fe	^40^Ar^16^O^+^
^75^As	^40^Ar^35^Cl^+^
